# Antigen presentation between T cells drives Th17 polarization under conditions of limiting antigen

**DOI:** 10.1016/j.celrep.2021.108861

**Published:** 2021-03-16

**Authors:** Viola L. Boccasavia, Elena R. Bovolenta, Ana Villanueva, Aldo Borroto, Clara L. Oeste, Hisse M. van Santen, Cristina Prieto, Diego Alonso-López, Manuel D. Diaz-Muñoz, Facundo D. Batista, Balbino Alarcón

**Affiliations:** 1 Interactions with the Environment Program, Centro Biología Molecular Severo Ochoa, Consejo Superior de Investigaciones Científicas, Universidad Autónoma de Madrid, 28049 Madrid, Spain; 2 Centro de Investigación del Cáncer, Instituto de Biología Molecular y Celular del Cancer, and Centro de Investigación Biomédica en Red de Cáncer (CIBERONC), CSIC-University of Salamanca, Campus Unamuno s/n, 37007 Salamanca, Spain; 3 Center for Physiopathology Toulouse-Purpan, INSERM UMR1043/CNRS UMR5282, CHU Purpan, BP3028, 31024 Toulouse, France; 4 Ragon Institute of MGH, MIT, and Harvard, Cambridge, MA 02139, USA

**Keywords:** antigen presentation by T cells, trogocytosis, Th17, Treg, limiting antigen, vaccine dosing

## Abstract

T cells form immunological synapses with professional antigen-presenting cells (APCs) resulting in T cell activation and the acquisition of peptide antigen-MHC (pMHC) complexes from the plasma membrane of the APC. They thus become APCs themselves. We investigate the functional outcome of T-T cell antigen presentation by CD4 T cells and find that the antigen-presenting T cells (Tpres) predominantly differentiate into regulatory T cells (Treg), whereas T cells that have been stimulated by Tpres cells predominantly differentiate into Th17 pro-inflammatory cells. Using mice deficient in pMHC uptake by T cells, we show that T-T antigen presentation is important for the development of experimental autoimmune encephalitis and Th17 cell differentiation *in vivo*. By varying the professional APC:T cell ratio, we can modulate Treg versus Th17 differentiation *in vitro* and *in vivo*, suggesting that T-T antigen presentation underlies proinflammatory responses in conditions of antigen scarcity.

## Highlights

• CD4 T cells take up antigen/MHC complexes and present them to cognate T cells
• After T-T cell antigen presentation, responding T cells differentiate into Th17
• T-T antigen presentation underlies Th17 generation in autoimmunity and viral infection
• Scarcity of antigen leads to Th17 differentiation, whereas abundance leads to Treg

## Graphical abstract

**Figure:**
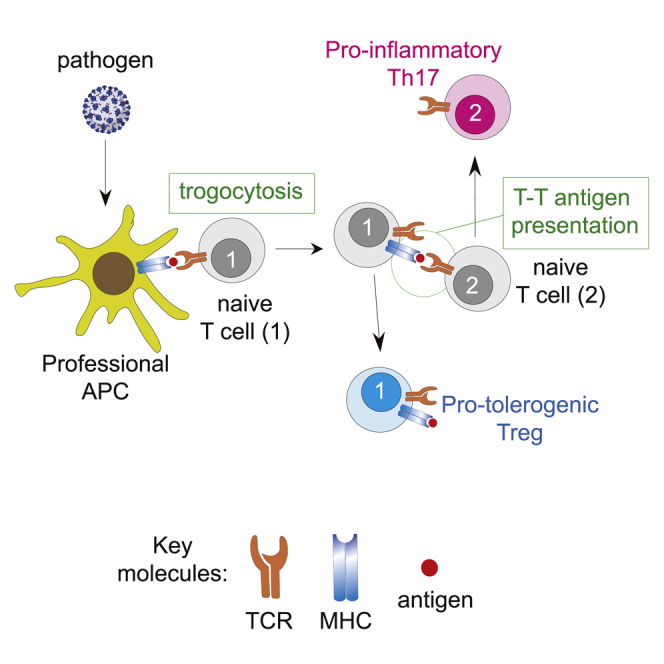

## Teaser

Boccasavia et al. show that T cells acquire antigen/MHC from professional antigen-presenting cells and convert themselves into presenting cells for other T cells of the same antigen specificity. Such T-T antigen presentation results in differentiation of the responding T cells into Th17 that mediate autoimmunity and responses to viral infections

## Introduction

T cells communicate through signaling membrane receptors triggered by soluble mediators such as hormones, chemokines, and cytokines, and by lipid bilayer-encapsulated mediators (Carlin et al., 2001), the most extensively studied being microvesicles or exosomes (Torralba et al., 2019). T cells release exosomes into the intercellular space of the immunological synapse (IS), containing proteins and nucleic acids, such as small interfering (siRNA), which act to modulate gene expression within the antigen-presenting cell (APC) and thus its function. Another important mechanism of intercellular communication within the immune system is trogocytosis (Joly and Hudrisier, 2003), where T cells and natural killer (NK) cells acquire micrometer-sized fragments of the APC membrane (Carlin et al., 2001; Vanherberghen et al., 2004). These fragments can contain immune-modulating membrane receptors such as killer inhibitory receptors and major histocompatibility complex (MHC) class I and II complexes. Previously it was shown that MHC-II vesicles are released by APCs and acquired by responding T cells (Arnold and Mannie, 1999; Patel et al., 1999). T cells also acquire peptide MHC (pMHC) complexes at the IS via a process that requires T cell receptor (TCR) internalization and TCR signaling (Huang et al., 1999; Hudrisier et al., 2001). In addition, they acquire CD80 and CD86, ligands of the co-stimulatory receptor CD28 (Hwang et al., 2000; Sabzevari et al., 2001). This could convert T cells into APCs. Both CD8 T cell-dendritic cell (DC) interactions and secondary T cell-T cell interactions are required to generate protective CD8 T cells (Gérard et al., 2013), and CD8 recall responses depend on CD8 T cells taking up MHC-II from DCs to present it to CD4 T cells and obtain their help (Romagnoli et al., 2013). In contrast, other results have suggested that pMHC acquisition by T cells may play an inhibitory role (Dhainaut and Moser, 2014). For instance, T cell antigen presentation to other T cells has been associated with the induction of anergy, apoptosis, and even tolerance (Chai et al., 1998; Mannie et al., 1996), and may represent a mechanism to limit clonal expansion (Tsang et al., 2003).

In previous work, we showed that T cells trogocytose APC membrane fragments through the IS in a TCR-driven and Rras2- and RhoG-dependent process (Martínez-Martín et al., 2011). This is accompanied by the internalization of MHC-II by the cognate T cells (Martínez-Martín et al., 2011). Here, we show that T cells express trogocytosed pMHC-II on their own membrane and, using RhoG-deficient T cells, demonstrate the relevance of these antigen-presenting T cells as APCs. The effector fate of the two T cells is completely different: antigen-presenting T cells differentiate into regulatory T cells, whereas T cells activated by T cell APCs differentiate into Th17. We propose that T-T antigen presentation is normally present in the immune response and that its balance is decisive for the activation of a pro-inflammatory response.

## Results

### T cells acquire and express MHC-II on their own surfaces in an antigen-dependent manner

**Figure 1:**
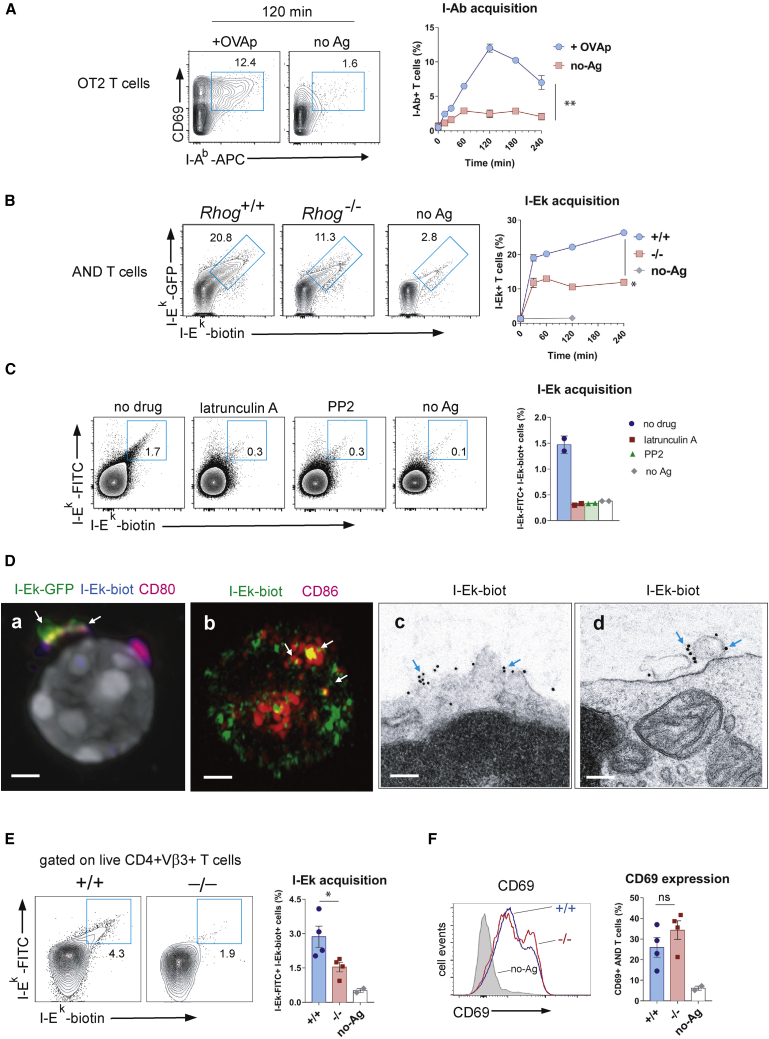
Trogocytic CD4 T cells acquire and display cognate MHC-II complexes together with CD28 ligands on their own plasma membrane (A) Time-dependent expression of I-A^b^ by OT2 TCR transgenic T cells upon incubation with untreated BMDCs (no-Ag) or BMDCs loaded with antigenic OVA peptide (ovalbumin 323–339, OVAp). Two-color contour plots show the expression of I-A^b^ and CD69 on gated CD4 T cells from mice of the indicated genotype. Insets indicate the percentage of I-A^b+^ CD69^+^ CD4 T cells. Quantification (means ± SEMs of triplicates) is shown in the graph to the right (^∗∗^p < 0.01, 2-tailed paired Student’s t test). (B) Time-dependent expression of I-E^k^ by AND CD4 T cells from b/b mice upon incubation with murine DCEK fibroblasts, transfected with the GFP-tagged I-E^k^α subunit and loaded with antigenic MCC peptide (moth cytochrome *c* 88-103; MCCp). AND CD4 T cells become double positive for GFP and a biotinylated anti-I-E^k^ antibody added to intact cells (left). Quantification (means ± SEMs of triplicates) is shown in the graph to the right (^∗^p < 0.05, 2-tailed paired Student’s t test). (C) Expression of I-E^k^ on the surface of AND CD4 T cells from b/b mice after incubation for 1 h with MCCp-loaded BMDCs from k/b mice, in the presence of 20 μM of the actin polymerization inhibitor latrunculin A or 20 μM of the Src tyrosine kinase inhibitor PP2. Quantification (means ± SEMs of duplicates) is shown in the bar graph to the right. (D) Expression of acquired I-E^k^ and CD80 on the cell surface of AND CD4 T cells from b/b mice after 1 h of incubation with DCEK cells, transfected with the GFP-tagged I-E^k^α subunit and loaded with MCCp. T cells were stained with biotin-labeled anti-I-E^k^ and Alexa 555-labeled anti-CD80 antibodies, as indicated, and analyzed by confocal microscopy (a midplane confocal section is shown in micrograph a, nucleus in gray). Micrograph b shows I-E^k^ and CD86 expression on the plasma membrane of AND T cells after incubation with MCCp-loaded BMDCs, analyzed by ELYRA super-resolution microscopy (a z axis projection of confocal sections). Analysis of I-E^k^ expression on AND T cells by electron microscopy (EM) after incubation with DCEK fibroblasts and purification of T cells and pre-embedding immunogold labeling with 10-nm streptavidin-gold particles (micrographs c and d). Blue arrows mark the presence of gold particles associated with the plasma membrane or with surface-bound microvesicles (micrographs c and d). Representative micrographs of 20–30 cells were taken for all techniques. (E) Flow cytometry analysis of the percentage of live anti-I-E^k^-FITC^+^ anti-I-E^k^-biotin^+^ Vβ3^+^ CD4 AND T cells in popliteal and inguinal lymph nodes 24 h after footpad immunization of AND WT and AND *Rhog*^*−/−*^ mice in k/b background with 100 μg MCCp and 10 μg LPS or LPS only (no-Ag). Quantification (means ± SEMs of quadruplicates) is shown in the bar plot to the right (^∗^p < 0.05, 2-tailed unpaired Student’s t test). (F) CD69 expression on Vβ3^+^ CD4 AND T cells isolated as in (E). Quantification (means ± SEMs of quadruplicates) is shown in the bar plots to the right. Gray histogram is a control of mice injected with LPS only (no-Ag) (ns, not significant, p > 0.05, 2-tailed unpaired Student’s t test).

We used two TCR transgenic mouse models to assay the expression of trogocytosed antigen/MHC (pMHC) complexes on T cells: the OT2 TCR, which recognizes a peptide derived from ovalbumin (OVAp) presented by the MHC-II allotype I-A^b^, and the AND TCR, which recognizes a peptide derived from moth cytochrome *c* (MCC) presented by the MHC-II allotype I-E^k^. We found that upon incubation with OVAp-loaded bone marrow-derived dendritic cells (BMDCs), OT2 CD4 T cells expressed I-A^b^ in a time-dependent manner (Figure 1A). The expression of I-A^b^ reached a maximum after 2 h of incubation and was higher in OT2 T cells that expressed the activation marker CD69 (Figure 1A). Even though activated mouse T cells do not transcribe MHC-II genes, we used AND TCR transgenic CD4 T cells to demonstrate that MHC-II on T cell plasma membranes is acquired from the APCs. AND CD4 T cells can be positively selected in the thymus both by I-A^b^ and by I-E^k^ (Kaye et al., 1992). We incubated purified AND CD4 T cells from mice in pure H-2^b^ background (b/b), which are unable to express I-E locus products (Mathis et al., 1983), with a DCEK cell line transfected with the I-E^k^α chain fused to GFP. Cell surface expression of I-E^k^ by AND CD4 T cells was determined by flow cytometry, following the acquisition of GFP and extracellular labeling with an anti-I-E^k^ antibody. We used RhoG-deficient AND CD4 T cells on a b/b background as a genetic control for TCR-triggered trogocytosis and MHC acquisition (Martínez-Martín et al., 2011). AND CD4 T cells expressed I-E^k^ in an antigen- and RhoG-dependent manner (Figure 1B), proving that they acquired I-E^k^ directly from the APC.

Antigen-dependent acquisition of I-E^k^ by wild-type (WT) AND CD4 T cells (b/b background), co-cultured with BMDCs (k/b background), was inhibited by the Src kinase and actin polymerization inhibitor2 PP2 and latrunculin A, respectively (Figure 1C). This further supports the notion that TCR triggering-dependent I-E^k^ acquisition is a trogocytic process, sharing characteristics with phagocytosis.

AND T cells also acquired the CD28 ligands CD80 and CD86 from BMDCs loaded with MCCp after an overnight incubation (Figure S1A). Cell surface display of CD80 by AND T cells was due to acquisition and not to endogenous transcription since qRT-PCR of AND T cells did not detect mRNA for CD80 (Figure S1B). The expression of CD28 ligands by AND T cells was also dependent on RhoG, suggesting that it was acquired from BMDCs by trogocytosis (Figure S1C). We studied the distribution of I-E^k^ and CD80 acquired from MCCp-loaded, I-E^k^-GFP-transfected DCEK by standard confocal microscopy. Acquired I-E^k^ was followed both by its GFP moiety and by extracellular labeling with anti-I-E^k^. We observed that I-E^k^ was not distributed homogenously on the T cell surface but formed large aggregates, some of which co-localized with CD80 (arrow, Figure 1D, micrograph a; Video S1). A similar experiment using ELYRA superresolution microscopy showed that I-E^k^ and CD86, acquired from MCCp-loaded BMDCs, distributed on the T cell surface in clusters of different sizes, some of which co-localized (arrows, Figure 1D, micrograph b; Video S2). Finally, we analyzed the organization of I-E^k^ clusters at the T cell surface by transmission electron microscopy. Upon external labeling of intact T cells with biotinylated anti-I-E^k^ and streptavidin coupled to 10-nm gold particles, we found I-E^k^ clusters both as integral membrane proteins (arrows, Figure 1D, micrograph c) and associated with membrane-attached microvesicles (arrows, Figure 1D, micrograph d; a section of an entire cell is shown in Figure S1D). We briefly addressed the intracellular trafficking of trogocytosed MHC-II and found that acquired intracellular I-E^k^ partly co-localized with Lamp-1^+^ and with CD63^+^ vesicles (Figure S1E), suggesting that MHC-II reaches the T cell membrane through multivesicular bodies. Further experimentation would be required to confirm this hypothesis.

Video S1. 3D reconstruction of confocal serial sections of a Tpres cell displaying I-Ek-GFP (in green), stained with biotinylated anti-I-Ek (in blue) and with anti-CD80 (in red), related to Figure 1

Video S2. 3D reconstruction of ELYRA serial sections of a Tpres cell stained with biotinylated anti-I-Ek (in green) and with anti-CD86 (in red), related to Figure 1

T cells also acquired bystander MHC-II complexes, but only when cognate antigen-loaded MHC-II was present. Thus, when OT2 T cells were incubated with BMDCs of k/b background loaded with OVAp antigen, the T cells became double positive for I-A^b^ and I-E^k^ (Figure S1F). The same effect was observed when AND T cells were incubated with BMDCs of k/b background loaded with MCCp. An explanation for this effect is that cognate and bystander MHC-IIs co-cluster at the immunological synapse and could be acquired together by the T cell (Krogsgaard et al., 2005).

We immunized WT and *Rhog*^*−/−*^ AND TCR transgenic mice on a k/k background with MCCp plus lipopolysaccharide (LPS) in the footpad and removed the draining popliteal lymph nodes 24 h later for analysis. We found that up to 4% of the Vβ3^+^ AND CD4 T cells became positive for I-E^k^, whereas RhoG deficiency reduced that percentage 2-fold (Figure 1E). Both WT and RhoG-deficient AND CD4 T cells were equally stimulated *in vivo*, as indicated by the expression of the CD69 marker (Figure 1F). Reduced expression of I-E^k^ by *Rhog*^*−/−*^ T cells *in vivo* was therefore not due to a differential exposure to antigen. These data indicate that CD4 T cells acquire and express MHC-II molecules *in vivo* and that RhoG plays an important role in that process.

### T cells that express acquired MHC-II activate cognate naive T cells

**Figure 2:**
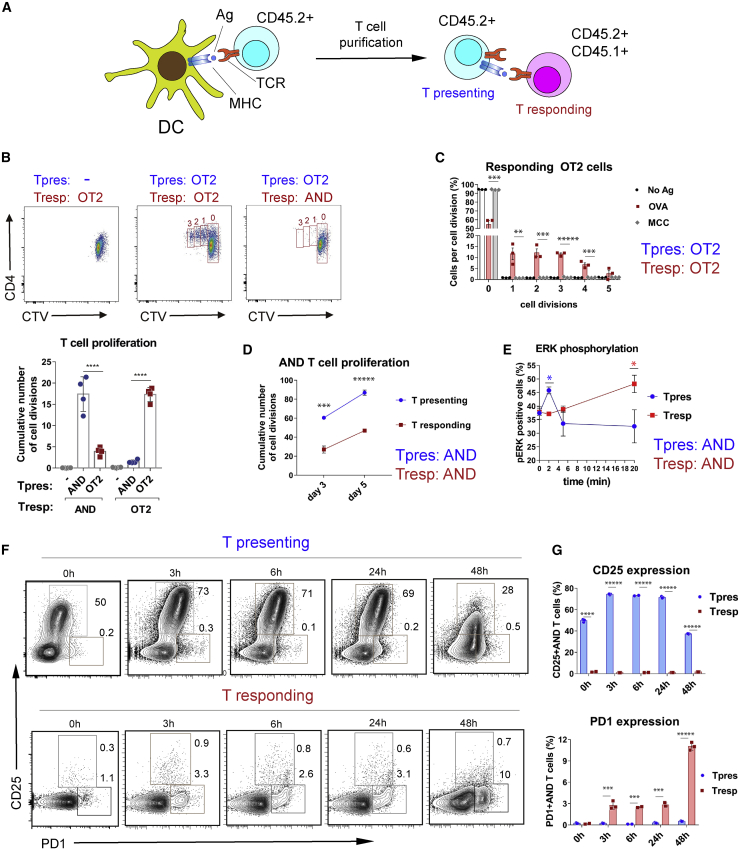
CD4 T cells that have trogocytosed and display MHC-II present antigen and stimulate other cognate naive T cells (A) Experimental setup. (B) Proliferation of naive OT2 and AND Tresp cells upon 3 days of co-culture with purified OT2 or AND Tpres cells previously exposed to BMDCs loaded with OVAp or MCCp, respectively. Fluorescence-activated cell sorting (FACS) plots show representative images of CTV-labeled OT2 and AND Tresp proliferation upon co-culture with or without OT2 Tpres cells. Bar graph shows quantification of AND and OT2 Tresp proliferation upon incubation with AND or OT2 Tpres cells (means ± SEMs of quadruplicates; ^∗∗∗∗^p < 0.0001 [2-tailed unpaired Student’s t test]). (C) Proliferation of OT2 Tresp upon 3 days of co-culture with purified OT2 Tpres cells, previously exposed to k/b BMDCs loaded with OVAp, MCCp, or no peptide (no-Ag). The number of cell divisions was calculated according to CTV dilution. The bar plot shows the means ± SEMs of triplicates.^∗∗^p < 0.01; ^∗∗∗^p < 0.001; ^∗∗∗∗∗^p < 0.00001 (2-tailed unpaired Student’s t test). (D) Cumulative number of cell divisions of CTV-labeled Tpres and Tresp AND T cells co-cultured for 3 and 5 days. The graph shows the means ± SEMs of quadruplicates. ^∗∗∗^p < 0.001; ^∗∗∗∗∗^p < 0.00001 (2-tailed unpaired Student’s t test). (E) Induction of ERK phosphorylation upon co-incubation of Tpres and Tresp AND T cells for the indicated time points. Line plots represent means ± SEMs (n = 3). ^∗^p < 0.05 (2-tailed unpaired Student’s t test). (F) Two-color contour plots showing the expression of CD25 and PD1 activation markers by CD4 AND Tpres and Tresp cells after co-incubation for the indicated time periods. Tpres cells were previously incubated overnight with MCCp-loaded BMDCs. Time point 0 h shows marker expression before co-incubation. (G) Quantification of data shown in (F). Bar plots represent means ± SEMs (n = 3). ^∗∗∗^p < 0.001; ^∗∗∗∗∗^p < 0.00001 (2-tailed unpaired Student’s t test).

To investigate the stimulatory capacity of T cells that have acquired MHC-II from APCs, we undertook a series of experiments in which purified naive T cells were co-cultured with antigen-loaded BMDCs and purified once more to remove the BMDCs (Figure S2A; from here called T presenting cells, or Tpres) before incubation with a second set of purified naive T cells (from here called Tresponding, or Tresp). To distinguish Tpres from Tresp cells during subsequent analysis, these cells were isolated from TCR transgenic mice expressing different combinations of the CD45.1 and CD45.2 alleles (Figures 2A and S2B).

OT2 cells were cultured overnight with BMDCs (k/b background) loaded with OVAp, purified (giving rise to Tpres), and cultured for 6 days with CellTrace Violet (CTV)-labeled naive OT2 T cells (Tresp). Tpres induced Tresp proliferation, as indicated by CTV dilution (Figure 2B). When OT2 Tpres cells were incubated with naive AND T cells, the latter did not proliferate or did so in a marginal manner. Purified AND T cells that had been incubated with BMDCs (k/b) loaded with MCCp (Tpres) stimulated the proliferation of naive AND T cells but not of OT2 T cells (Figure 2B), and OT2 Tpres cells pre-incubated with MCCp-loaded BMDCs were unable to induce the proliferation of OT2 Tresp (Figure 2C). We measured the capacity of AND Tpres cells to induce the proliferation of AND Tresp cells in comparison with their own proliferative response. Tpres and Tresp cells proliferated in a time-dependent manner, although the proliferative response of Tresp cells was lower (Figure 2D). As a very early event of T cell activation, we investigated the phosphorylation of ERK induced upon co-incubation of Tpres and Tresp AND T cells. Co-culture induced a very transient increase in ERK phosphorylation in Tpres cells that peaked at t = 5 min, whereas ERK phosphorylation was induced more slowly in Tresp cells, with a maximum at t = 20 min or later (Figure 2E). These results indicate that trogocytic T cells acquire and display MHC-II complexed to their cognate antigenic peptide and can stimulate cognate naive T cells. However, whereas most Tpres cells were CD25^+^, Tresp cells did not express much of the marker, even after 48 h of co-incubation (Figure 2F). By contrast, Tresp but not Tpres cells rapidly upregulated expression of the T cell activation marker PD1, peaking at the longest time of co-incubation (48 h; Figure 2G). These results provided further evidence that Tpres cells can activate Tresp cells although a different set of activation markers is induced.

### Tpres cells are enriched in Treg and Tresp cells in Th17

**Figure 3:**
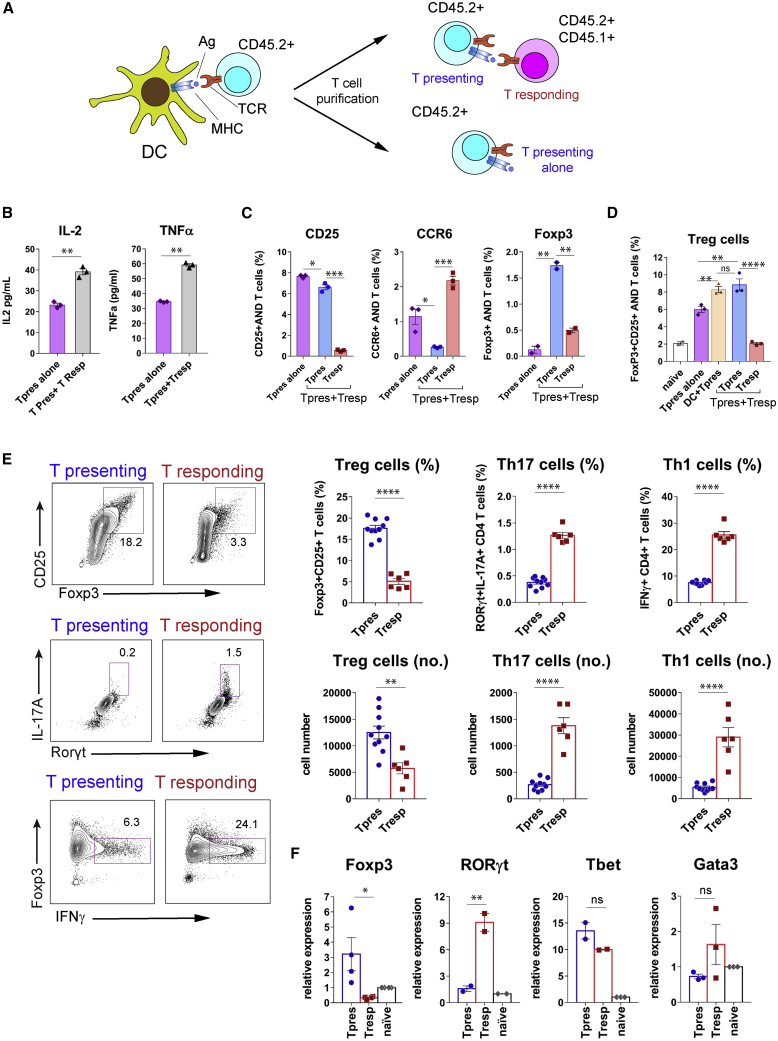
Tpres cells are enriched in Treg and Tresp cells in Th17 (A) Experimental setup. After purification by cell sorting, AND Tpres cells are incubated for 3–6 days in the absence or presence of naive AND Tresp cells. (B) ELISA measurement of IL-2 and TNF-α concentrations in supernatants of Tpres cells cultured for 3 days in the presence or absence of Tresp cells. The bar graphs show the means ± SEMs of triplicates.^∗∗^p < 0.01 (2-tailed unpaired Student’s t test). (C) Expression of CCR6, CD25, and Foxp3 by Tpres and Tresp cells from the experiment in (B), measured by flow cytometry. Bar graphs represent means ± SEMs (n = 3). ^∗^p < 0.05; ^∗∗^p < 0.01; ^∗∗∗^p < 0.001 (2-tailed unpaired Student’s t test). (D) Generation of FoxP3^+^CD25^+^ T cells within AND Tpres and Tresp cell populations. After overnight incubation with MCCp-loaded BMDCs and purification, Tpres were cultured alone (Tpres alone) or together with naive AND Tresp cells (Tpres+Tresp) for 6 days and then analyzed by flow cytometry for Foxp3 and CD25 expression. In parallel, AND T cells were cultured uninterrupted for 6 days with MCC-loaded BMDCs (DC+Tpres). Data represent the means ± SEMs of biological triplicates. ^∗∗^p < 0.01; ^∗∗∗∗^p < 0.0001; ns, not significant (2-way ANOVA test). (E) Tpres and Tresp cells were stained with surface CD25 and intracellular IL-17A, Foxp3, RORγt, or IFNγ. Two-color contour plots are on the left and quantification in the bar plots to the right. ^∗∗^p < 0.01; ^∗∗∗∗^p < 0.0001 (2-tailed unpaired Student’s t test). (F) Quantification via qRT-PCR of mRNA expression of sorted Tpres, Tresp cells, and naive AND T cells. Data are presented as the means ± SEMs of n = 2–4 biological replicates normalized to the mean values of naive cells (set as 1). ^∗^p < 0.05; ^∗∗^p < 0.01 (2-tailed unpaired Student’s t test).

We next assessed how interaction between Tpres and Tresp cells mutually influenced their differentiation into effector cells by culturing purified AND Tpres cells (Figure S2C) alone or together with AND Tresp cells (Figure 3A). Interleukin-2 (IL-2) and tumor necrosis factor α (TNF-α) concentrations in the culture supernatant increased by almost 2-fold when Tpres cells were co-cultured with Tresp cells as opposed to the Tpres cells alone (Figure 3B). As observed above, the expression of CD25 by Tpres cells was only slightly affected by a 3-day co-culture with Tresp cells, while Tresp cells again expressed very little CD25 (Figure 3C). We also found marked differential expression of chemokine receptor CCR6, a marker for Th17 cells (Hirota et al., 2007) and the Treg master regulator Foxp3. Tpres cells downregulated CCR6 when they were co-cultured with Tresp cells compared with Tpres cultured in isolation, whereas Tresp cells upregulated the expression of CCR6. The opposite effect was detected for Foxp3 expression: Tpres cells upregulated Foxp3 expression when co-cultured with Tresp cells, whereas the percentage of Foxp3^+^ cells within the Tresp population was almost 4-fold lower (Figure 3C). The upregulation of Foxp3 in Tpres cells when co-incubated with Tresp cells was as high as in Tpres cells cultured for 6 days with BMDCs plus antigen (Figure 3D).

We verified the lineage commitment of Tpres and Tresp upon co-culture by the detection of key transcription factors via antibody staining and gene expression studies. AND Tpres were purified by cell sorting after overnight incubation with BMDCs (k/b) plus MCCp and subsequently co-cultured with AND Tresp cells. After 6 days of co-culture, a clear population of CD25^+^Foxp3^+^ cells was found within Tpres cells, but less so within the Tresp population (Figure 3E). Conversely, the presence of IL-17A^+^ CD4 T cells that expressed the master regulator of Th17 differentiation, RORγt, was more than 3-fold higher within the Tresp than within the Tpres population (Figure 3E). Tresp cells were also better producers of interferon γ (IFNγ) than Tpres cells (Figure 3E). Analysis by qRT-PCR of the expression of the key regulators of Treg, Th17, Th1, and Th2 differentiation showed that Tresp expressed significantly more RORγt and less Foxp3 than Tpres (Figure 3F), thus confirming their preferential differentiation toward Th17 and Treg, respectively. By contrast, there were no significant differences in the expression of Tbet and Gata-3, master regulators of Th1 and Th2 cells, respectively (Figure 3F).

To link the differentiation of Tpres cells into Treg with the acquisition of MHC-II (as shown in Figure 1), we studied whether acquired I-E^k^ was still detected on the surface of Tpres cells after 6 days of co-culture. Approximately 20% of Tpres cells were I-E^k+^, whereas only a few Tresp cells were detected as positive (Figure S3A). Furthermore, the I-E^k+^ population within Tpres was enriched in Foxp3 and CD25 Treg markers (Figure S3B). This finding indicates that trogocytic T cells that have acquired MHC-II are the ones differentiating into Treg. The role of antigen specificity in Th17 induction by T-T antigen presentation was resolved by culturing MCC- and OVA-loaded BMDCs derived from k/b mice with AND T cells (k/k background) and subsequently co-culturing the purified Tpres cells with either naive AND T cells or naive OT2 T cells as Tresp cells (Figure S4A). AND Tresp cells differentiated into Th17 but OT2 T cells did not (Figure S4B), demonstrating that Th17 differentiation of Tresp cells is specific for the antigen recognized by Tpres cells. A possible explanation for the antigen selectivity of Tresp cell differentiation into Th17 in spite of the fact that trogocytic T cells also acquire bystander MHC-II (Figure S1F) is that the latter is not enriched in a particular antigen peptide.

We next investigated Tpres and Tresp differentiation *in vivo*. CD45.2^+^ AND T cells stimulated *ex vivo* with BMDCs loaded with MCCp were inoculated into CD45.1^+^ mice that had been previously inoculated with naive CD45.2^+^CD45.1^+^ AND T cells (Figure S4C). Six days later, the expression of Treg markers CD25 and Foxp3 and Th17 markers IL-17A and CCR6 was analyzed within the Tpres (CD45.2^+^) and Tresp (CD45.2^+^CD45.1^+^) CD4 T cell populations. The results showed a preferential differentiation of Tpres cells into Treg and a preferential differentiation of Tresp into Th17 *in vivo* (Figure S4D).

### Genome-wide analysis of gene transcription confirms Treg and Th17 differentiation within Tpres and Tresp cells, respectively

**Figure 4:**
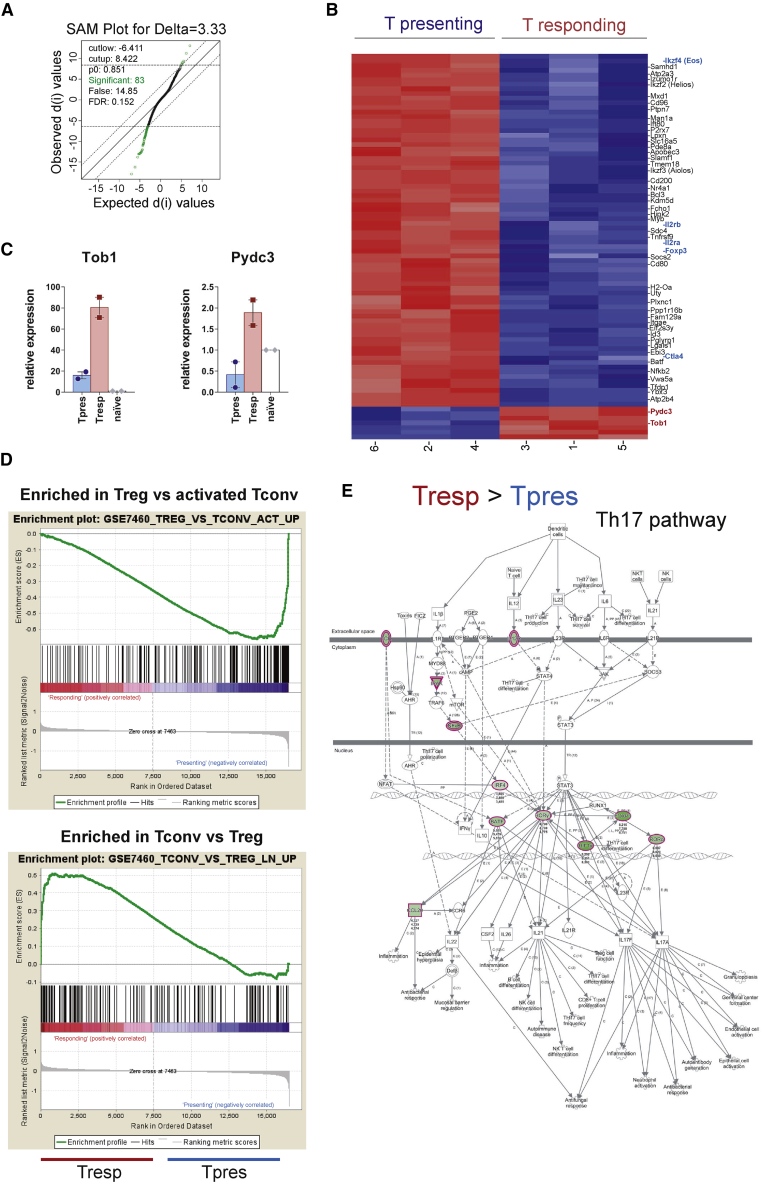
Total gene expression analysis reveals signatures of Treg in Tpres and Th17 in Tresp cells (A) Significance analysis of microarray (SAM) plot diagram showing the comparison of transcriptomes from Tpres and Tresp FACS-sorted cells isolated at day 5 of co-culture. Transcripts differentially and not differentially expressed between the 2 cell types using a false discovery rate (FDR) of 0.152 are depicted as green and black circles, respectively. A total of 83 transcripts showed statistically significant variations between both cell types. (B) Heatmap representation of genes differentially transcribed in Tpres versus Tresp cells analyzed in biological triplicates. Red indicates the highest expression; dark blue the lowest. Genes with a functional implication in Treg are highlighted in bold blue type. Genes associated with Th17 function are highlighted in bold red type. (C) qRT-PCR analysis of Tob1 and Pydc3 gene expression in Tpres and Tresp cells after 5 days of co-culture. Bar plots show the means ± SEMs (n = 2 mice per group). Expression values are normalized to those of naive CD4 AND T cells (set as 1). (D) Gene set enrichment analysis (GSEA) from the Treg versus activated T conventional cell and from the T conventional versus Treg signatures from the Broad GSEA mSig database shows an enrichment for Treg in Tpres and for conventional T cells in Tresp. (E) The top score IPA Th17 pathway. Green-colored shapes are more highly expressed in Tresp than in Tpres. Different shapes represent the molecular classes of the proteins: kinases are shown as triangles, membrane receptors as double ellipses, transcriptional regulators as single ellipses, and cytokines and chemokines as squares. Direct and indirect interactions are indicated by solid and dashed lines, respectively.

We further investigated the distinct differentiation pathways of AND Tpres and Tresp cells by co-culturing them for 6 days and preparing total RNA from each T cell population after cell sorting. Microarray-based whole-genome transcriptional analysis showed that only 83 genes were differentially expressed in Tpres versus Tresp, with a false discovery rate (FDR) of 0.152 (Figure 4A; Table S1). Most of the 83 genes were more highly expressed in Tpres than in Tresp cells (Figure 4B). Among those, we found Ikzf4 (Eos), IL2rb, IL2ra (CD25), Foxp3, and Ctla4, genes that have been associated with Treg function (Rieder and Shevach, 2013; Rodríguez-Perea et al., 2016). Only 7 genes were found to be expressed significantly higher in Tresp than in Tpres, and only 2 of those, Tob1 and Pydc3, encoded for proteins of known function (Figure 4B). qRT-PCR assays confirmed the upregulation of Tob1 and Pydc3 in Tresp compared to Tpres cells (Figure 4C). Tob1 is an anti-proliferative gene that plays an important role in Th17 function by controlling its expansion. In fact, Tob1 downregulation has been associated with a higher severity of disease in patients with multiple sclerosis (Baranzini, 2014). Pydc3 is a component of the NLRP3 inflammasome that has been shown to play a T cell-intrinsic role in Th17 differentiation in experimental autoimmune encephalitis (EAE) as well as in the regulation of Th17 differentiation in patients with rheumatoid arthritis (Martin et al., 2016; Zhao et al., 2018). Activation of the NLRP3 inflammasome could be behind the differentiation of Tresp toward Th17 since an Ingenuity Pathway Analysis (IPA) of the microarray data showed an exacerbation of this pathway in Tresp (Figure S5A). Further analysis of the microarray data showed an enrichment in Treg genes within the Tpres population (gene set enrichment analysis [GSEA]; Figure 4D), whereas IPA showed the upregulation of the Th17 pathway in Tresp (Figure 4E). IPA also showed a fingerprint of the Th1 pathway in Tresp cells and of the Th2 pathway in Tpres (Figure S5B). In agreement with this, we found that Tresp cells produced more IFNγ than did Tpres cells (Figure 3E). Furthermore, trogocytic T cells (Tpres in the present article) have been shown to differentiate into Th2 effector cells (Reed and Wetzel, 2019).

### RhoG-deficient mice show an attenuated response in a Th17-driven disease model

**Figure 5:**
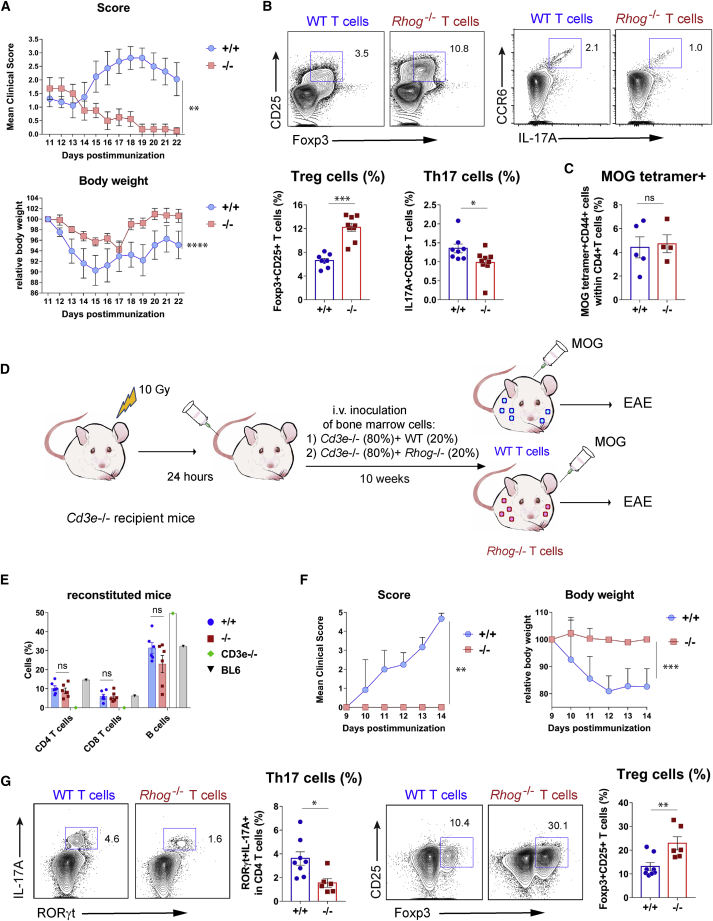
Deficiency in T-T antigen presentation leads to reduced Th17/Treg ratios and resistance to EAE (A) Evolution of neurological symptoms (score) and body weight in MOG-immunized WT and *Rhog*^*−/−*^ mice. Neurological scores were according to Borroto et al. (2016) Graphs represent the means ± SEMs (n = 8–9 mice per group; paired 2-tailed Student’s t test [weight] and non-parametric matched-pairs signed rank Wilcoxon 2-tailed test [score]). ^∗∗^p < 0.01; ^∗∗∗∗^p < 0.0001. (B) Two-color contour plots showing expression of Foxp3, CD25, IL-17A, and CCR6 in WT and *Rhog*^*−/−*^ mice sacrificed at day 22 (A). Bar graphs show the percentages of Foxp3^+^CD25^+^ and IL17A^+^CCR6^+^ T cells (means ± SEMs; n = 8–9 mice per group; unpaired 2-tailed Student’s t test; ^∗^p < 0.05; ^∗∗∗^p < 0.001). (C) WT and *Rhog*^*−/−*^ mice were immunized with MOG, and the draining popliteal lymph nodes were collected 7 days later. The presence of MOG-reactive T cells was analyzed by flow cytometry on the CD4^+^CD44^+^ activated population by incubation with I-A^b^ OVA_329-337_ tetramer. Bar plot shows the means ± SEMs (n = 4–5 mice per group; unpaired 2-tailed Student’s t test. ns, not significant). (D) Experimental setup of the bone-marrow adoptive transfer experiment. (E) BM reconstitution was tested in the blood of chimeric mice before immunization with MOG. Bar plots show the percentage (means ± SEMs) of CD4 T, CD8 T, and B cells within the white blood cell population in bone marrow chimeras and, as reference, in *Cd3e*^*−/−*^ and WT C57BL/6 mice. (F) Score and body weight evolution in BM chimeras reconstituted with either WT or *Rhog*^*−/−*^ T cells. Graphs represent the means ± SEMs (n = 6–8 mice per group; 2-tailed Student’s t test [weight] and non-parametric matched-pairs signed rank Wilcoxon 2-tailed test [score]; ^∗∗^p < 0.01; ^∗∗∗^p < 0.001). (G) Two-color contour plots showing the expression and the percentages of Foxp3 and CD25 Treg markers and of the IL-17A and RORγt Th17 markers in WT and *Rhog*^*−/−*^ BM chimeras sacrificed at day 14 (F). Bar plots show the means ± SEMs (n = 6–8 mice per group; unpaired 2-tailed Student’s t test; ^∗^p < 0.05; ^∗∗^p < 0.01).

Since Tpres cells preferably differentiate toward Treg and Tresp cells toward Th17, we investigated the relevance of T-T antigen presentation *in vivo* in a myelin oligodendrocyte glycoprotein antigen (MOG)-induced model of EAE. A number of genetic studies support the idea that Th17 cells play an important role in EAE pathology, whereas Treg are protective (Rostami and Ciric, 2013). We compared the response to MOG immunization in *Rhog*^*−/−*^ mice deficient in trogocytosis and therefore in the process of pMHC acquisition and presentation (Figure 1; Martínez-Martín et al., 2011) with that of WT mice. Immunization with MOG resulted in the development of symptoms of neurological impairment in WT mice, as well as loss of body weight, whereas *Rhog*^*−/−*^ mice had much milder symptoms (Figure 5A). These findings correlated with the increased abundance of Th17 and reduced Treg frequency in WT mice compared to *Rhog*^*−/−*^ mice (Figure 5B). In contrast, the percentage of CD4 T cells specific for MOG antigen, detected by staining with an MOGp(I-A^b^) tetramer, in *Rhog*^*−/−*^ mice was indistinguishable from their WT counterparts, suggesting that T cells in *Rhog*^*−/−*^ mice are not deficient in their response to MOG antigen (Figure 5C). These data are consistent with the idea that the capacity of CD4 T cells to acquire and present antigen to cognate T cells is important for Th17 differentiation and EAE development.

We assessed a T cell-intrinsic role of RhoG via bone marrow reconstitution experiments, lethally irradiating *Cd3e*^*−/−*^ mice, which lack T cells (DeJarnette et al., 1998), and reconstituting them with a mixture of 20% bone marrow cells derived from either WT or RhoG-deficient mice and 80% *Cd3e*^*−/−*^ bone marrow cells. Since bone marrow precursors from *Cd3e*^*−/−*^ mice generate all types of hematopoietic cells except T cells, the reconstituted *Cd3e*^*−/−*^ mice will express RhoG in all cells, including 80% of their hematopoietic cells, except in T cells, all of which will lack RhoG (Figure 5D). Bone marrow reconstitution resulted in the similar presence of CD4 and CD8 T cells in lymphoid tissues of mice reconstituted with WT and *Rhog*^*−/−*^ donors (Figure 5E). Immunization with MOG led to severe neurological impairment in mice reconstituted with WT, but not in those reconstituted with RhoG-deficient T cells (Figure 5F). The presence of Th17 and Treg cells was analyzed post-mortem in the cervical lymph nodes by staining with CD25 and Foxp3 (as Treg markers) and IL-17A together with RORγt (as Th17 markers). The results showed a higher abundance of Th17 cells in mice with WT T cells, which are able to present antigen to other T cells, than in mice whose T cells lack RhoG and are defective in T-T antigen presentation (Figure 5G). In contrast, Treg cells were more abundant in *Rhog*^*−/−*^ mice. Resistance of *Rhog*^*−/−*^ mice to EAE may not be due to defective T-T antigen presentation but to an effect of RhoG on T cell maturation in the thymus or during T cell activation. However, the percentage and number of thymocytes according to their distribution in the major DN, DP, CD4SP, and CD8SP populations is not altered in *Rhog*^*−/−*^ versus WT mice (Figure S6A). Furthermore, according to the expression of CD69 and CD5 markers in DP thymocytes (Figure S6B) and the expression of Nur77 (Figure S6C), *Rhog*^*−/−*^ mice are not deficient in either positive or negative selection. In addition, WT and *Rhog*^*−/−*^ OT2 T cells induced CD25 expression and proliferation to the same extent in response to varying concentrations of OVAp *in vitro* (Figure S6D). Lastly, the *ex vivo* proliferative response to MOG stimulation of CD4 T cells isolated from draining lymph nodes of MOG-immunized mice was not affected by RhoG deficiency (Figure S6E). These data plus the tetramer data (Figure 5C) indicate that the resistance to EAE is not due to an unforeseen role of RhoG in T cell maturation and activation but rather to a deficiency in T-T antigen presentation and subsequent differentiation into Th17 cells. These results suggest that Th17 differentiation *in vivo* is linked to the capacity of T cells to acquire and express antigen/MHC to other T cells and that such Th17 cells are functionally involved in the development of EAE.

We extended these *in vivo* studies beyond TCR-transgenic T cells by inoculating OT2 Tpres cells into recipient mice with a polyclonal T cell repertoire (Figure S7). Recipient OVAp-I-A^b^ tetramer^+^ Tresp cells showed significant enrichment in Th17 cells that depended on a functional *Rhog* gene in Tpres cells.

### Role of DC abundance in Treg versus Th17 differentiation

**Figure 6:**
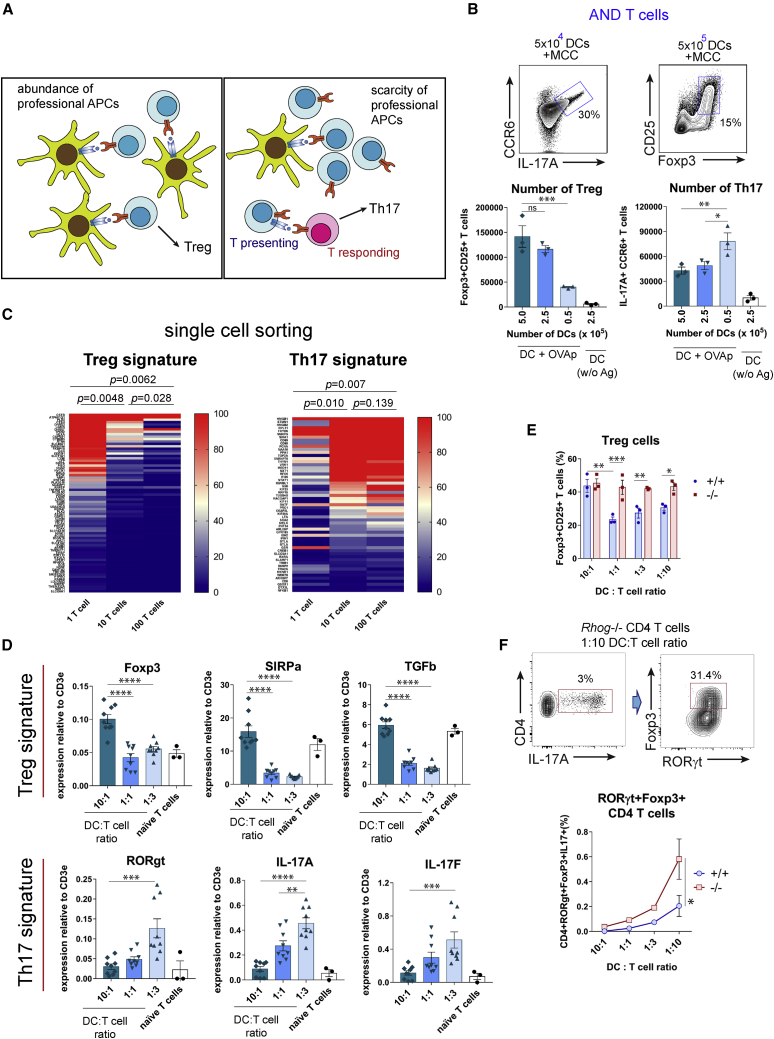
The DC:T cell ratio determines Treg versus Th17 differentiation *in vitro* (A) Graphical representation of working hypothesis on the effect of professional APC:T cell ratio on CD4 differentiation. (B) Generation of Treg and Th17 cells upon 6-day co-culture of 2.5 × 10^6^ AND T cells with varying numbers of MCCp-loaded BMDCs. Treg versus Th17 differentiation was determined according to the expression of CD25, Foxp3, IL-17A, and CCR6 markers. Two-color contour plots show Th17 and Treg differentiation under the optimal DC doses. Bar graphs show the total number of cells with the Treg and Th17 phenotype (means ± SEMs of triplicate datasets; ^∗^p < 0.05; ^∗∗^p < 0.01; ^∗∗∗^p < 0.001; 2-tailed unpaired Student’s t test). (C) Heatmap representation of genes differentially transcribed in conditions of 1, 10, or 100 AND T cells per well. Plotted genes correspond to those that have been associated with either Treg or Th17 signatures after GSEA analysis (Figure S9). Color-coded relative number of reads per gene is indicated in the scale bars to the right. Statistical analysis of expression differences was carried out by a 2-tailed paired Student’s t test. p values are considered significant if <0.05. (D) qRT-PCR analysis of genes associated with a Treg signature (Foxp3, SIRPα, and TGF-β) or a Th17 signature (RORγt, IL-17A, and IL-17F) after mRNA extraction from AND T cell and MMCp-loaded BMDC co-cultures at indicated DC:T cell ratios. Bar plots show the means ± SEMs of n = 3–9 biological replicas. mRNA expression was normalized to the expression of the T cell-restricted CD3ε gene. ^∗∗^p < 0.01; ^∗∗∗^p < 0.001; ^∗∗∗∗^p < 0.0001 (1-way ANOVA test). (E) Differentiation of AND T cells from WT and *Rhog*^*−/−*^ mice according to the DC:T cell ratio after 6 days of co-culture. Bar plots show the mean ± SEM of n = 3–9 biological replicas. mRNA expression is shown relative to that of CD3ε used to normalize for T cell number. ^∗^p < 0.05; ^∗∗^p < 0.01; ^∗∗∗^p < 0.001 (2-tailed unpaired Student’s t test). (F) Co-expression of RORγt and Foxp3 in *Rhog*^*−/−*^ AND T cells co-cultured for 6 days with MCCp-loaded BMDCs at a 1:10 DC:T cell ratio. The graph shows the percentage of AND WT and *Rhog*^*−/−*^ CD4^+^IL-17A^+^ cells that co-express RORγt and Foxp3 upon co-culture at different DC:T cell ratios. Data are shown as means ± SEMs of triplicate cultures. ^∗^p < 0.05 (2-tailed paired Student’s t test).

The likelihood of a naive T cell to be stimulated by an antigen-bearing DC versus an antigen-bearing Tpres, thereby determining its fate, should depend on the relative frequency of antigen-presenting DCs versus cognate T cells (Figure 6A). We cultured a constant number of AND CD4 T cells with different numbers of MCCp-loaded BMDCs and analyzed their differentiation toward Treg and Th17 six days later. The number of Foxp3^+^CD25^+^ CD4 Treg was directly proportional to the number of antigen-presenting DCs, while the number of Th17 was inversely proportional (Figure 6B). The same effect was detected for OT2 CD4 T cells co-cultured with varying numbers of OVAp-loaded BMDCs or splenic CD8^+^ DCs (Figure S8). The abundance of professional APCs appeared to favor Treg fate, whereas their scarcity favors Th17 fate, presumably because T-T antigen presentation is more likely if naive T cells have not previously encountered a professional APC.

In a complementary cell sorting-based approach, we co-cultured 10 antigen-loaded DCs with 1, 10, or 100 T cells per well, reasoning that with a single T cell per well, no T-T antigen presentation would occur, whereas this would be favored with 10 or 100 T cells per well. After 6 days, cells from wells grown under the same condition were pooled in 3 biological replicates before mRNA extraction and gene expression analysis by RNA sequencing (RNA-seq) (Table S2). GSEA analysis of the 3 types of samples revealed fingerprints of higher Treg content in the samples of 1 T cell per well than in the others and, conversely, more fingerprints of high Th17 content in the 10 and 100 T cell per well conditions (Figure S9). Simultaneous comparison of all gene sets indicating Treg and Th17 signatures (Table S3) showed that the Treg signature was more represented in the 1 T cellper well condition than in the 10 T cell per well condition, and in the latter than in the 100 T cell per well condition (Figure 6C). An inverse trend was observed for the Th17 signature (Figure 6C). Although key genes such as Foxp3 or RORγt were not identified during sequencing (Table S3), qRT-PCR analysis of mRNAs for genes involved in Th17 and Treg differentiation or function showed a direct correlation between the ratio of DC APCs and T cells for the Foxp3, signal regulatory protein α (SIRPα) and transforming growth factor β (TGF-β) Treg markers and an inverse correlation for the RORγt, IL-17A, and IL-17F Th17 markers (Figure 6D). The downregulation of Foxp3 with a decreasing DC:T cell ratio was impaired in RhoG-deficient AND T cells compared to their WT counterparts, suggesting that such downregulation is dependent on T-T antigen presentation (Figure 6E). Interestingly, decreasing DC:T cell ratios led to a deficient downregulation of Foxp3 within the IL-17A^+^RORγt^+^ CD4 T cell population when AND T cells lacked RhoG (Figure 6F). These data suggest that high ratios of T cells:professional APCs lead to enrichment in T-T antigen presentation and CD4 T cell differentiation toward Th17.

**Figure 7:**
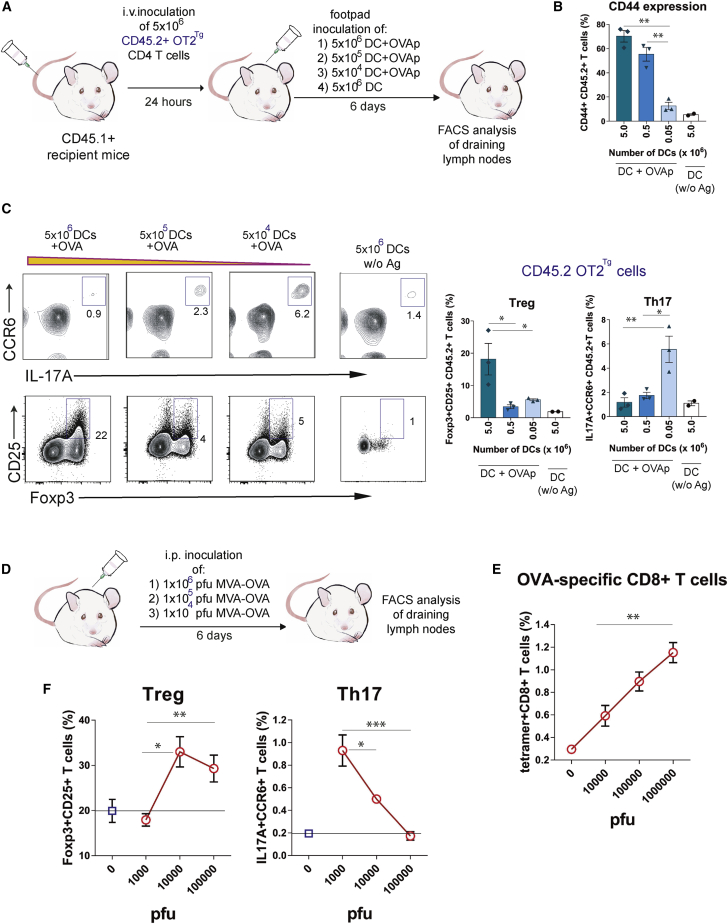
Abundance of DCs and antigen determines Treg versus Th17 response *in vivo* (A) Experimental setup of the immunization protocol with different numbers of DCs. (B) OT2 CD4 response to antigen was evaluated by measuring the expression of the CD44 activation marker as a function of the dose of OVAp-loaded DCs. Bar graphs show the means ± SEMs (n = 3 mice per condition; unpaired 2-tailed Student’s t test; ^∗∗^p < 0.01). (C) Two-color contour plots showing percentages of Foxp3^+^CD25^+^ Treg cells and IL-17A^+^CCR6^+^ Th17 cells within the CD45.2^+^CD4^+^ OT2 cell population in the function of the dose of DC APCs. Bar plots show the means ± SEMs (n = 3 mice per condition; unpaired 2-tailed Student’s t test; ^∗^p < 0.05; ^∗∗∗^p < 0.001). (D) Experimental setup of intraperitoneal (i.p.) infection with increasing number of plaque-forming units (PFUs) of MVA-OVA. (E) CD8 T cell response to the virus was evaluated by measuring the percentage of I-A^b^-OVAp tetramer^+^ cells within the CD8^+^ T cell population. Graph shows the means ± SEMs (n = 2–4 mice per condition; unpaired 2-tailed Student’s t test; ^∗∗^p < 0.01). (F) Quantification of the percentage of Foxp3^+^CD25^+^ Treg and IL-17A^+^CCR6^+^ Th17 within the splenic endogenous CD4 T cell population. Graphs show the means ± SEMs (n = 2–4 mice per condition; unpaired 2-tailed Student’s t test; ^∗^p < 0.05; ^∗∗^p < 0.01; ^∗∗∗^p < 0.001).

Similar effects were detected *in vivo*. We transferred naive CD45.2^+^ OT2 CD4 T cells into CD45.1^+^ mice and immunized these mice by footpad injection of different numbers of BMDCs previously loaded with OVAp. Six days after immunization, the popliteal and inguinal draining lymph nodes were removed, and T cell activation was analyzed after gating on the CD45.1^−^ OT2 CD4 T cell population (Figure 7A). The percentage of OT2 T cells that expressed the activation marker CD44 was directly proportional to the number of BMDCs used for immunization (Figure 7B). Likewise, the percentage of CD4 T cells with the Treg phenotype was highest in mice immunized with the highest number of BMDCs, whereas differentiation toward Th17 was more efficient in mice immunized with fewer BMDCs (Figure 7C). We also infected mice with different doses of the modified virus Ankara (MVA) encoding OVA (Figure 7D). MVA is a vaccinia variant that is unable to replicate in mouse cells *in vivo* and only gives rise to a single round of infection (El-Gogo et al., 2007). The number of infected APCs and the dose of antigen available should therefore be directly dependent on the dose of virus administered. The size of the responding H-2K^b^-OVA tetramer^+^ CD8 T cell population was directly proportional to the administered dose of MVA-OVA (Figure 7E). The percentage of CD4 T cells with Treg markers increased with the dose of virus, reaching a plateau at the intermediate dose, but the percentage of CD4 T cells with Th17 markers was significantly higher in mice infected with the lowest dose than in those infected with the intermediate and higher ones (Figure 7F). Together with the results above, a scenario appears in which an abundance of professional APCs favors interaction with naive T cells, which will differentiate toward Treg, whereas scarcity of APCs will allow T-T cell interaction, leading to Th17 differentiation.

## Discussion

We show that CD4 T cells acquire MHC-II from professional APCs and display it in an antigen-driven manner to other T cells. Using a combination of AND TCR transgenic T cells and APCs that can be unequivocally distinguished by their MHC haplotypes, we demonstrate that antigen/MHC-II complexes expressed by CD4 T cells are of external origin. This confirms previous studies showing that CD4 T cells take up not only MHC-I and MHC-II and display them on the cell surface but also ligands of CD28 (Arnold and Mannie, 1999; Huang et al., 1999; Hudrisier et al., 2001; Hwang et al., 2000; Patel et al., 1999; Sabzevari et al., 2001). We show that acquired MHC-II and CD80 form clusters at the CD4 T cell plasma membrane, potentially enabling T cells to become APCs themselves. We demonstrate here that this is indeed the case. Surprisingly, we found that Tpres and Tresp cells can influence each other and induce opposing differentiation fates where Tpres are enriched for Treg and Tresp are enriched for Th17. In addition, Tresp cells express features of Th1, whereas Tpres cells express those of Th2. The differential fate of both T cells after T-T antigen presentation is not just a consequence of experimental conditions *in vitro*, since inoculating mice with CD4 Tpres cells leads to differentiation toward Th17 of cognate T cells bearing a transgenic TCR.

A pending issue is to understand the signaling differences that make Tpres and Tresp cells follow distinct differentiation fates. From our results, it is clear that not only Tpres cells activate Tresp but also that Tresp influences Tpres cells in terms of IL-2, TNF-α, CCR6, or Foxp3 expression. We hypothesize that Tresp and Tpres cells compete for antigenic stimulation and use of cytokines, which ultimately affects the differentiation phenotype. Another unresolved issue is to understand how different a T-T cell immunological synapse is from a DC:T cell synapse and whether the accumulation of pMHC taken from the professional APC in large clusters, together or not with CD28 ligands, has an impact on T cell fate. While the first T cell must bind and “collect” pMHC complexes on the surface of the professional APC, the second, Tresp cell contacts the Tpres cell with already concentrated pMHC complexes. Along this line, we show that trogocytic T cells acquire and express both the cognate pMHC-II complex and the bystander MHC-II. However, trogocytic Tpres cells only activate their cognate counterparts. We explain this conundrum by suggesting that unlike the cognate acquired pMHC-II complex, which is enriched with the cognate peptide antigen, the bystander MHC-II should not be enriched in any particular peptide.

It is somehow surprising that we obtain up to 20% Tregs in our pure T cell cultures in the absence of added polarizing cytokines. Although we cannot provide an answer, an interesting possibility is that TGF-β, which we showed to be produced by Tpres cells during T-T antigen presentation, acts in an autocrine loop to favor differentiation toward Treg by upregulating Foxp3 expression (Chen and Konkel, 2010). Also, trogocytic T cells have a more sustained TCR signaling than non-trogocytic T cells (Reed and Wetzel, 2019), and this could be a determinant for the differentiation of trogocytic cells toward Th2 as previously suggested (Reed and Wetzel, 2019). We speculate that in the presence of Tresp cells, this could determine differentiation toward Treg. An additional consideration is that Tresp cells could also take up and trogocytose membrane fragments from the Tpres cell and again recycle pMHC complexes on their own plasma membrane. Such a process has not yet been evaluated, but if it were to occur, it could lead to a ceaseless cycle of trogocytosis and T-T antigen presentation.

We have gathered evidence that *in vivo* inoculation of CD4 Tpres cells leads to the expansion of cognate T cells within the endogenous polyclonal repertoire and their differentiation into Th17. Since it is estimated that between 20 and 200 naive CD4 T cells of the same peptide antigen specificity are present in the lymph nodes and spleen of non-immunized mice (Moon et al., 2007), it is theoretically possible that a Tpres cell may encounter and activate another cognate naive CD4 T cell, thus inducing its differentiation toward Th17. We have addressed this possibility by showing that mice bearing RhoG-deficient T cells are both resistant to the development of EAE and deficient in the generation of Th17. As RhoG is required for the acquisition by trogocytosis of MHC-II (Martínez-Martín et al., 2011; the present article) and RhoG-deficient T cells have not been found to be defective during thymic development or in the response to antigen *in vitro* and *in vivo* (the present article), we conclude that the process of T-T antigen presentation is relevant for the generation of Th17 and the induction of EAE.

The Treg/Th17 differentiation dichotomy as a function of the antigen dose could explain the well-known effects of high antigen doses. It has long been known that the administration of high doses of antigen can suppress the immune response to the same antigen (Mitchison, 1964). This phenomenon could drive the tolerogenic effect of large organ (e.g., liver) transplantation (Cunningham et al., 2013), and the therapeutic outcome of high-dose allergen exposure (Soyer et al., 2013). Interestingly, the tolerogenic effect of high doses of allergen has been associated with the activation of Treg (Soyer et al., 2013).

Our data show that T-T antigen presentation at low antigen doses favors Th17 versus Treg differentiation. We also found that T-T presentation favors Th1 versus Th2 differentiation, although in our experiments the differences did not reach statistical significance. However, older studies correlated mid-range antigen doses with IFNγ production and Th1 differentiation and high antigen doses with IL4 production and Th2 differentiation (Hosken et al., 1995), suggesting again that low concentrations rather than high concentrations of antigen are proinflammatory.

Since pathogens do not usually breach skin or mucosal barriers in large quantities, T-T antigen presentation could have emerged as a way to recruit more antigen-specific T cells than those directly activated by a low-level infection or limiting antigen-loaded professional APCs, simultaneously unleashing an inflammatory response before the pathogen has replicated to potentially tolerogenic levels. In this regard, the titration of vaccine dose is known to be essential to achieve a protective cellular response, in which excessive antigen is detrimental (Leggatt, 2014). In addition, low antigen dose has been shown to selectivity induce CD4 T cells with higher functional avidity and protective efficacy (Billeskov et al., 2017). Here, we provide a mechanistic explanation for the development of future low-dose antigen-based strategies, which may be relevant in face of the present coronavirus disease 2019 (COVID-19) pandemic.

## STAR★Methods

### Key Resources Table

**Table:** 

REAGENT or RESOURCE	SOURCE	IDENTIFIER
**Antibodies**		

Rat anti-mouse CD4-AlexaF647(clone RM4-5)	BD Biosciences	Cat #557681, RRID:AB_396791
Rat anti-mouse CD4-PE (clone RM4-5)	BD Biosciences	Cat #553049, RRID:AB_394585
Rat anti-mouse CD4-PerCP (clone RM4-5)	BD Biosciences	Cat #553052, RRID:AB_394587
Rat anti-mouse CD4-FITC (clone RM4-5)	BD Biosciences	Cat #553047, RRID:AB_394583
Rat anti-mouse CD4-biotin (clone RM4-5)	BD Biosciences	Cat #553045, RRID:AB_394581
Rat anti-mouse CD4-V450 (clone RM4-5)	BD Biosciences	Cat #560468, RRID:AB_1645271
Mouse anti-mouse CD45.2-APC (clone 104)	BD Biosciences	Cat #558702, RRID:AB_1645215
Mouse anti-mouse CD45.1-APC-Cy7(clone A20)	BD Biosciences	Cat #560579, RRID:AB_1727487
Mouse anti-mouse CD45.1-biotin (clone A20)	BD Biosciences	Cat #553774, RRID:AB_395042
Mouse anti-mouse CD45.1-BV421 (clone A20)	BD Biosciences	Cat #563983, RRID:AB_2738523
Mouse anti-mouse I-E(k)-FITC (clone 17-3-3)	BD Biosciences	Cat #558846, RRID:AB_397140
Mouse anti-mouse I-E(k)-biotin (clone 17-3-3)	BD Biosciences	Cat #558845, RRID:AB_397139
Mouse anti-mouse I-A(b)-biotin (clone AF6-120.1)	Biolegend	Cat #116404, RRID:AB_313723
Hamster anti-mouse Vβ3-biotin (clone KJ25)	BD Biosciences	Cat #553207, RRID:AB_394707
Hamster anti-mouse CD69-FITC (clone H1.2F3)	BD Biosciences	Cat #557392, RRID:AB_396675
Hamster anti-mouse CD69-PE (clone H1.2F3)	BD Biosciences	Cat #553237, RRID:AB_394726
Hamster anti-mouse CD279 (PD1)-FITC (clone J43)	eBioscience	Cat #11-9985-85, RRID:AB_465473
Rat anti-mouse Vα2 TCR-PE (clone B20.1)	BD Biosciences	Cat #553289, RRID:AB_394760
Rat anti-mouse CD25-FITC (clone PC61)	Biolegend	Cat #102006, RRID:AB_312855
Rat anti-mouse CD25-PerCP (clone PC61)	Biolegend	Cat #102028, RRID:AB_2295974
Rat anti-mouse CD86-PE-Cy5 (clone GL1)	eBiosciences	Cat # 15-0862-82, RRID:AB_468778
Rat anti-mouse CD11b-PerCP-Cy5.5 (clone M1/70)	BD Biosciences	Cat # 550993, RRID:AB_394002
Rat anti-mouse CD11b-biotin (clone M1/70)	BD Biosciences	Cat #553309, RRID:AB_394773
Rat anti-mouse F4/80-Biotin (clone BM8)	Biolegend	Cat #123106, RRID:AB_893501
Hamster anti-mouse CCR6-PE-Cy7 (clone 29-2L17)	Biolegend	Cat #129816, RRID:AB_2072798
Rat anti-mouse IL17A-AF647 (clone TC11-18H10)	BD Biosciences	Cat #560224, Cite this (BD Biosciences Cat# 560224
Rat anti-mouse IL17-PerCP-Cy5.5 (clone TC11-18H10)	BD Biosciences	Cat #560666, RRID:AB_1937311
Rat anti-mouse Foxp3-PE (clone NRRF-30)	eBiosciences	Cat #12-4771-82, RRID:AB_529580
Hamster anti-mouse CD80-AlexaF647 (clone 16-10A1)	Biolegend	Cat #104718, RRID:AB_492824
Hamster anti-mouse CD80-PE (clone 16-10A1)	BD Biosciences	Cat #553769. RRID:AB_395039
Mouse anti-mouse RORγt - BV421 (clone Q31-378)	BD Biosciences	Cat #562894, RRID:AB_2687545
Mouse anti-mouse H-2Kb-PE (clone AF6-88.5)	BD Biosciences	Cat #553570, RRID:AB_394928
Mouse anti-mouse H-2Kk-FITC (clone 36-7-5)	BD Biosciences	Cat #553592, RRID:AB_394941
Hamster anti-mouse CD11c (clone HL3)	BD Biosciences	Cat #553800, RRID:AB_395059
Rat anti-mouse CD16/32 Purified (clone 2.4G2)	BD Biosciences	Cat #553142, RRID:AB_394657
Rat anti-mouse Gr1 -Biotin (clone RB6-8C5)	BD Biosciences	Cat #553125, RRID:AB_394641

**Bacterial and virus strains**		

Modified Vaccine Ankara (MVA)-OVA	El-Gogo et al., 2007	N/A
Mycobacterium Tuberculosis H37 Ra Strain	Difco	N/A

**Chemicals, peptides, and recombinant proteins**		

DMEM	CBMSO Facility	N/A
RPMI	CBMSO Facility	N/A
L-Glutamine	CBMSO Facility	N/A
Penicillin and Streptomycin	CBMSO Facility	N/A
B-mercaptoethanol	Sigma-Aldrich	Cat #M3148
Sodium pyruvate	CBMSO Facility	N/A
Non-essential amino acids	CBMSO Facility	N/A
Fetal Bovine Serum HyCLone	Fisher Scientific	Cat #12389802
Bovine Serum Albumin	Rockland	Cat #BSA-1000
Paraformaldehyde	Sigma-Aldrich	Cat #158127
OVA peptide 323-339: ISQAVHAAHAEINEAGR	ANASpec	Cat #AS-27024
MCC peptide 88-103: ANERADLIAYLKQATK	CBMSO Facility	N/A
MOG peptide 35-55: MEVGWYRSPFSRVVHLYRNGK	Spikem	Cat #EPK1
Recombinant Murine GM-CSF	Peprotech	Cat #315-03
Poly-L-lysine hydrobromide	Sigma-Aldrich	Cat #P1274
CellTrace Violet Cell Proliferation kit	Thermo Fisher	Cat #C34571
CellTrace CFSE Cell Proliferation kit	Thermo Fisher	Cat #C34570
Neomycin trisulfate salt hydrate	Sigma-Aldrich	Cat #N6386
Pertussis toxin	Sigma-Aldrich	Cat #P7208
Freund’s complete adjuvant	Sigma-Aldrich	Cat #F5881
PMA	Sigma-Aldrich	Cat #P1585
Ionomycyne	Sigma-Aldrich	Cat # I0634
Brefeldine A	eBioscience	Cat #00-4506-51
Trizol	Thermo Fisher	Cat # 15596026
CountBright Absolute Counting Beads	Thermo Fisher	Cat #C36950
Dynabeads M-280 Streptavidin	Thermo Fisher	Cat #11206D
AMPure XP beads	Beckman Coulter	Cat #A63882
Mowiol Dabco	Calbiochem	N/A
Epon Embedding Medium kit	Sigma-Aldrich	Cat #45359

**Critical commercial assays**		

Naive CD4+ T Cell Isolation Kit, mouse (MACS)	Miltenyi Biotec	Cat #130-104-453
Foxp3 Transcription Factor Staining Buffer Set	Thermo Fisher	Cat #00-5523-00
RNeasy Plus Mini Kit	QIAGEN	Cat #74134
RNeasy Micro Kit	QIAGEN	Cat #74004
SuperScrip III First-Strand Synthesis SuperMix for qRT-PCR	Thermo Fisher	Cat #11752250
GoTaq® qPCR Master Mix	Promega	Cat #A6002
GeneChip WT PLUS Reagent Kit	Affymetrix	Cat #902280
GeneChip Mouse Gene 2.0 ST Array	Affymetrix	Cat #902118
Monarch Total RNA Miniprep Kit	New England Biolabs	Cat #T2010S
NEBNext Single Cell/Low Input RNA Library Prep Kit for Illumina	New England Biolabs	Cat #E6420S

**Experimental models: cell lines**		

Mouse: Fibroblast cell line expressing I-Ek and CD80	Laboratory of Ronald Germain	N/A

**Experimental models: organisms/strains**		

Mouse: *Rhog−/−* in C57BL/6 background	Vigorito et al., 2004	N/A
Mouse: AND TCR transgenic	Kaye et al., 1989	N/A
Mouse: OT2 TCR transgenic	Hogquist et al., 1994	N/A
Mouse: *Cd3e−/−* in C57BL/6 background	The Jackson Laboratory	JAX: 004177
Mouse: CD45.1+ Ly5.1 mouse	Charles River Laboratories	Strain code: 494

**Oligonucleotides**		

Primer for Foxp3 Forward: GCCTACAGTGCCCCTAGTCA	This paper	N/A
Primer for Foxp3 Reverse: TTGAGGGAGAAGACCCCAGT	This paper	N/A
Primer for IL-17A Forward: AACATGAGTCCAGGGAGAGC	This paper	N/A
Primer for IL-17A Reverse: GAGGTAGTCTGAGGGCCTTC	This paper	N/A
Primer for RORγt Forward: TAGCACTGACGGCCAACTTA	This paper	N/A
Primer for RORγt Reverse: TCGGAAGGACTTGCAGACAT	This paper	N/A
Primer for SIRPα Forward: CTCTCCCCGGAATATCACCC; Reverse: ACAGGTTAGCAATCCCACGA	This paper	N/A
Primer for SIRPα Reverse: ACAGGTTAGCAATCCCACGA	This paper	N/A
Primer for TGFβ1t Forward: CGTCAGACATTCGGGAAGCA	This paper	N/A
Primer for TGFβ1t Reverse: TGCCGTACAACTCCAGTGAC	This paper	N/A
Primer for IL-17F Forward: ATGAAGTGCACCCGTGAAAC	This paper	N/A
Primer for IL-17F Reverse: TCTGGAATTCACGTGGGACA	This paper	N/A
Primer for CD3e Forward: AACACTTTCTGGGGCATCCT	This paper	N/A
Primer for CD3e Reverse: ATGTTCTCGGCATCGTCCT	This paper	N/A
Primer for Tbet Forward: CTGGAGCCCACAAGCCATTA	This paper	N/A
Primer for Tbet Reverse: CCCCTTGTTGTTGGTGAGCT	This paper	N/A
Primer for GATA3 Forward: GCAACCTCTACCCCACTGTG	This paper	N/A
Primer for GATA3 Reverse: CCCATTAGCGTTCCTCCTCC	This paper	N/A
Primer for Tob1 Forward: ACTTTTGCTGCCACCAAGTT	This paper	N/A
Primer for Tob1 Reverse: GAGCTACCTTGCTGCTACGG	This paper	N/A
Primer for Pydc3 Forward: TGCTCACTCACTCACTGCTT	This paper	N/A
Primer for Pydc3 Reverse: AGGTCATGGTTCAGTAAGGAC	This paper	N/A

**Software and algorithms**		

Flowjo analysis software	FlowJo	N/A
Diva	BD Biosciences	N/A
Imaris	Bitplane	N/A
ImageJ	NIH	N/A
GraphPad Prism 7	GraphPad	N/A
Zen software	Zeiss	N/A

**Other**		

FACSCanto II Cell Analyzer	BD Biosciences	Cat #338962
2100 Bioanalyzer Instrument	Agilent	Cat #G2939BA
Complete GeneChip® Instrument System	Affymetrix	Cat #00-0213; #00-0218; #00-0362; #00-0186
NextSeq 500 System	Illumina	Cat # SY-415-1002
NextSeq 500/550 v2.5 Kits	Illumina	Cat #20024906
CFX384 Touch Real-Time PCR Detection System	BioRad	Cat #1855485
LSM 780 microscope	Zeiss	N/A
Elyra PS.1 microscope	Zeiss	N/A
UC6 ultramicrotome	Leica	N/A

### Resource availability

#### Lead contact

Further information and requests for resources and reagents should be directed to and will be fulfilled by the Lead Contact, Balbino Alarcón (balarcon@cbm.csic.es).

#### Materials availability

This study did not generate new unique reagents.

#### Data and code availability

The published article includes all datasets (Table S1. Microarray gene expression analysis of Tpres (samples 2, 4, and 6) and Tresp (samples 1, 3, and 5), related to Figure 4, Table S2. RNA-seq data of T cell cultures at the indicated number per well, related to Figure 6, Table S3. Aggregated gene signatures of Treg and Th17 differentiation obtained after pooling GSEA data of Figure S9, related to Figure 6) generated and analyzed during this study.

### Experimental model and subject details

#### Mice

*Rhog*^−/−^ mice were established in a C57BL/6 background and were generated as described in Vigorito et al. (2004). These mice were crossed with mice transgenic for the AND TCR (Vα11.1/Vβ3) specific for an MCC peptide presented by I-E^k^ (Kaye et al., 1989). *Rhog*^−/−^ were also crossed with mice transgenic for the OT2 TCR (Vα2/Vβ5) specific for peptide 323-339 of chicken ovalbumin presented by I-A^b^ (Barnden et al., 1998). C57BL/6 mice bearing the pan-leukocyte marker allele CD45.1 were kindly provided by Dr. Carlos Ardavín (CNB, Madrid). *Cd3e*^−/−^ mice (DeJarnette et al., 1998), deficient in the expression of CD3ε were obtained from Jackson Laboratories. Mice were bred and maintained under SPF conditions in the animal facility of the Centro de Biología Molecular Severo Ochoa with unlimited access to food and water. All experiments were carried out in strict accordance with the European Commission legislation for the protection of animal used for scientific purposes (2010/63/EU).

#### Cell preparation and purification

DCEK is a cell line derived from fibroblasts transfected with plasmids encoding I-Ek and CD80. These cells were cultured in DMEM with 10% fetal bovine serum (FBS) supplemented with 2Mm L-Glutamine, 100 U/ml penicillin and 100 U/ml streptomycin. Bone marrow dendritic cells (DC) were generated as described (Lutz et al., 1999). Briefly, cells from mouse bone marrow were incubated with recombinant murine granulocyte-macrophage colony stimulating factor (RM GM-CSF 20ng/ml) for 10 days, changing the medium every 3 days. Phenotypic characteristics were assessed by flow cytometry on day 10 (CD11b^+^, CD11c^+^, CD80^+^, CD86^+^, H-2Kb^+^, H-2Kk^+^,Gr1^-^, F4/80^-^) to confirm proper maturation. Primary mouse CD4^+^ T cells were obtained from single-cell suspensions of lymph nodes (LN) and spleens from 5-8 week-old mice. The cells were homogenized with 40 μm strainers and washed in phosphate-buffered saline (PBS) containing 2% (vol/vol) fetal bovine serum (FBS). Spleen cells were resuspended for 5 minutes in AcK buffer (0.15 M NH_4_Cl, 10 mM KHCO_3_, 0.1 mM EDTA, pH7.2-7.4) to lyse erythrocytes and washed in PBS supplemented with 2% FBS. For *in vitro* cultures, cells were maintained in RPMI with 10% FBS supplemented with 2mM L glutamine, 100 U/ml penicillin, 100 U/ml streptomycin, 20 mM B-mercaptoethanol and 10 mM sodium pyruvate and 1% non-essential amino acids. For culture and *in vitro* assays, T cells from lymph nodes were either positively selected by sorting or negatively selected using CD4 T cell isolation kit (Macs Miltenyi Biotec; 130-104-453) or a combination of biotinylated antibodies followed by and incubation with streptavidin beads (Dynabeads, Invitrogen) for 30 min and separated using a Dynal Invitrogen Beads Separator.

#### Experimental autoimmune encephalomyelitis

Chronic EAE was induced in female C57BL/6 mice (6 to 8 week-old, 20 g body weight) by subcutaneously injecting a total of 150 μg of MOG 35–55 (Espikem) emulsified in Freund’s complete adjuvant (Sigma-Aldrich) and supplemented with Mycobacterium tuberculosis (H37Ra strain from Difco) at 1 mg/ml into both femoral regions. Mice were immediately injected intraperitoneally with 200 ng of pertussis toxin (Sigma-Aldrich) and, again, 48 hours after immunization. Animals were examined daily for clinical signs of disease, which were scored as follows: 0, no symptoms; 1, limp tail; 2, weakness of hind legs; 3, complete paralysis of hind legs; 4, complete hind leg and partial front leg paralysis; 5, moribund state (Choi et al., 2015).

#### Bone marrow reconstitution and adoptive transfer

8 week-old female mice were transferred into neomycin-supplemented water (Sigma) one week before the beginning of the procedure. They were lethally irradiated using 10 Gy, and injected intravenously 24h later with 5-10.10^6^ donor bone marrow cells. Mice were kept with neomycin-supplemented water up to 2 weeks after irradiation to prevent the development of any kind of infections. Animals were bled after 5 weeks to check for reconstitution efficiency. They were used for further experimentation 8 to 10 weeks after adoptive transfer.

### Method details

#### Flow cytometry

Cells were incubated with anti-CD16/32 in PBS, 1% BSA, 0.02% sodium azide before labeling with saturating amounts of the indicated fluorochrome-labeled or biotinylated mAbs, and fluorochrome-labeled streptavidin when necessary, for 20 min at 4°C. Lymph node and spleen cells were first stained in PBS with live/dead Fixable Near-IR Dead cell Stain kit. For transcription factor staining, cells were fixed with the fixation buffer (eBioscience Foxp3 / Transcription Factor Staining Buffer Set) and then stained with the specific intracellular antibodies in permeabilization buffer overnight at 4°C, and then washed. For intracellular staining, cells were stimulated for 2 h in 50 ng/mL PMA and 1 μg/mL ionomycin followed by an additional incubation for 4 h in Brefeldin A (eBioscience). Cells were then fixed and permeabilized before staining with the appropriate antibodies. Labeled cells were analyzed on a FACSCanto II flow cytometer (Becton Dickinson) and data was analyzed with FlowJo software (TreeStar). Counting of total cells was performed with CountBright ™ beads (Life Technologies).

#### Gene expression analysis

Tpres and Tresp cells were sorted in a FACSVantage sorter (Becton Dickinson) and RNA was extracted with an RNAeasy kit (QIAGEN 74134) according to the manufacturer’s instructions. RNA integrity was assessed using an Agilent 2100 Bioanalyzer (Agilent). Labeling and hybridizations were performed according to protocols from Affymetrix. Briefly, 100 ng of total RNA were amplified and labeled with the WT Plus reagent kit (Affymetrix) and then hybridized to Mouse Gene 2.0 ST Array (Affymetrix). Washing and scanning were performed using an Affymetrix *GeneChip* System (GeneChip Hybridization Oven 645, GeneChip Fluidics Station 450 and GeneChip Scanner 7G). Robust microarray analysis (RMA) algorithm was used for background correction, intra- and inter-microarray normalization, and expression signal calculation. Once the absolute expression signal for each gene was calculated in each microarray, a method called significance analysis of microarray (SAM) (Tusher et al., 2001) was applied to calculate significant differential expression. The method uses permutations to provide robust statistical inference of the most significant genes and provides P values adjusted to multiple testing using false discovery rate (FDR). Gene Set Enrichment Analyses were performed using GSEA v 2.2.2 (Subramanian et al., 2005) and hallmark and immunological signature collection of gene sets.

#### RNA-seq

Library preparation and sequencing were carried out in ‘Fundación Parque Científico de Madrid’. Briefly: Monarch Total RNA Miniprep Kit (New England BioLabs) was used for total RNA extraction following the manufacturer recommendations (including DNasa treatment).

Once extracted, 100 pg of total RNA from each sample were used as input for library preparation with “NEBNext Single Cell/Low Input RNA Library Prep Kit for Illumina” (New England BioLabs) following the manufacturer recommendations. The so-obtained libraries were validated and quantified in a 2100 Bioanalyzer (Agilent) and an equimolecular pool was made, purified using AMPure XP beads (Beckman Coulter) and titrated by quantitative PCR using the “Kapa-SYBR FAST qPCR kit forLightCycler480” and a reference standard for quantification. The library pool was denatured and seeded on a NextSeq v2.5 flowcell (Illumina) where clusters were formed and sequenced using a “NextSeq 500 High Output kit v2.5” (Illumina) in a 1x75 single-read sequencing run on a NextSeq 500 sequencer (Illumina).

#### RT-qPCR

Total RNA was extracted from T cell culture cells using the RNeasy Micro Kit (QIAGEN; 74004) or trizol extraction method. RNA obtained was reverse-transcribed using Superscript III reverse transcription kit (Invitrogen; 11752), and finally, the reverse-transcribed RNA was amplified with the appropriate primers listed in Table S1. All primers were designed to span at least one intron. Real-time qPCR analysis was performed using GoTaq qPCR Master Mix (Promega; A6002) in a CFX384 Touch Real-Time PCR Detection System Lightcycler (Bio Rad). Each mRNA value was normalized to CD3e mRNA and expressed as the relative RNA abundance.

#### Light microscopy

Cells were transferred to coverslips previously treated for 2 hours at RT or overnight at 4°C with 50 μg/ml Poly-L-lysine. The APCs (DCEK) are plated the day before on coverslips placed in wells of a p24 plate (16 mm) at 50x10^3^ cells per well and incubated in DMEM 10% FBS supplemented with 10 mM MCC peptide ON. Before putting them in contact with the T cells, they are washed with PBS to remove excess peptide. In order to obtain T cells, we collected the lymph nodes from AND mice and resuspended to a concentration of 2x10^6^ cells/ml. In each well of the p24 we added 250 μL and we incubated the cells at different time points. For short times (less than 10 min), a pulse is given to the p24 plate at 60xg for 20 s. For immunofluorescence assays, cells were plated onto slides previously coated with poly-L-Lysine for 2 hours at RT or overnight at 4°C (50 μg/ml), incubated for 30 min, washed in TNB buffer (100 mM Tris-HCl, pH 7.4. 150 mM NaCl, 2% BSA) fixed with 4% paraformaldehyde (PFA) in PBS for 30 min, blocked in PBS with 5%BSA and stained with the indicated primary antibodies followed by secondary antibodies. After staining, coverslips were washed twice and fixed onto glass slides with Mowiol/Dabco (Calbiochem). The samples were left to dry at RT for 24 hours and stored at 4°C afterward.

Confocal imaging was performed on a Zeiss LSM 780 microscope with a plan apochromat 20X, NA 0.8 objective for tissue sections or a plan apochromat 63X, NA 1.40 objective for other applications. Images were analyzed with Imaris (Bitplane) and ImageJ (NIH) softwares.

Structured Illumination Microscopy was performed on an Elyra PS.1 microscope (Carl Zeiss) using 488 and 640 nm laser excitation and a 63 × /1.40 plan apochromat oil-immersion objective (Zeiss). Two-color alignment was performed after each experiment day using a multicolor bead sample (Zeiss) and the channel alignment function in the Zen software (Zeiss). Images were reconstructed using Zen software with a theoretical point-spread function and a noise filter setting of –4.0 for both channels, which gave a good compromise between resolution and signal-to-noise ratio of the reconstructed images. Under these conditions, the lateral resolution was found to be 150 nm using 40-nm green fluorescent beads.

#### Electron microscopy

Processing of cells for TEM and imaging was performed by initially fixing with PFA 2%, for 20-30 min in PBS at room temperature and centrifuged at 11,300xg for four minutes. CD4+ GFP+ cells were incubated with Ab anti-IEk biotin in PBS + 1% BSA for 30 min RT and afterward with gold particles PAG of 15 nm 1.50 in a volume of 200 μl. A further step of fixation with 500 mL of 4% PFA + 2% GT BP 0.1M pH 7.4 for two hours has been performed. The pellet obtained, embedded in gelatin matrix was then cut in little cubes in order to proceed with Epon embedding. Blocks were sectioned (UC6 ultramicrotome; Leica), picked up on Formvar®-coated slot grids and post-stained with lead citrate. Sections were imaged using a transmission electron microscope (TEM).

### Quantification and statistical analysis

Quantitative data are shown as the means ± SEM. In each Figure legend, the *n* number refers to the number of animals or biological replicas. Two sets of data were considered significant with a p < 0.05 or lower. All experiments were carried out by comparing mice of the same age and sex, raised at the same location of the animal house and differing only in the indicated genotype. Decision on the number of mice needed to reach significance was built on previous experience for this and other papers. A parametric Student t test was used for most data and a Wilcoxon test for non-parametric data; one-way and two-way ANOVA tests were also used when comparing different variables. All data was analyzed using the GraphPad Prism 7 software.

## Supplemental information

Document S1. Figures S1–S9

Table S1. Microarray gene expression analysis of Tpres (samples 2, 4, and 6) and Tresp (samples 1, 3, and 5), related to Figure 4

Table S2. RNA-seq data of T cell cultures at the indicated number per well, related to Figure 6

Table S3. Aggregated gene signatures of Treg and Th17 differentiation obtained after pooling GSEA data of Figure S9, related to Figure 6

Document S2. Article plus supplemental information
