# Supplementary material for: Antigen presentation between T cells drives Th17 polarization under conditions of limiting antigen
Source: Cell Rep. 2021 Mar 16;34(11):108861. doi: 10.1016/j.celrep.2021.108861 (PMC7972993; doi:10.1016/j.celrep.2021.108861)
Supplement: Document S1. Figures S1–S9 [file mmc1.pdf]

Cell Reports, Volume *34*

Supplemental information

Antigen presentation between T cells drives Th17 polarization under conditions of limiting antigen

Viola L. Boccasavia, Elena R. Bovolenta, Ana Villanueva, Aldo Borroto, Clara L. Oeste, Hisse M. van Santen, Cristina Prieto, Diego Alonso-López, Manuel D. Diaz-Muñoz, Facundo D. Batista, and Balbino Alarcón

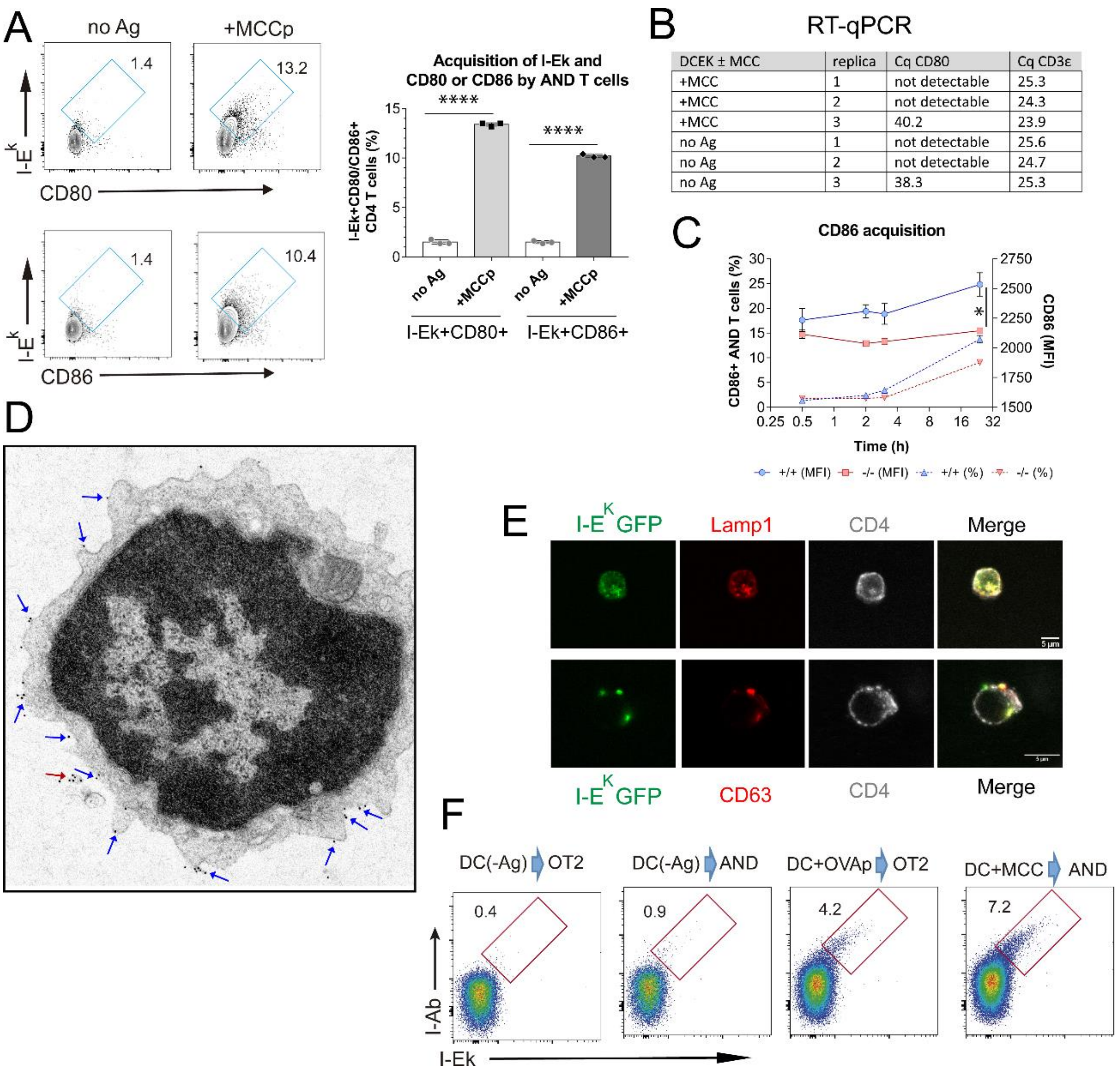

DCEK ± MCC	replica	Cq CD80	Cq CD3ε
+MCC	1	not detectable	25.3
+MCC	2	not detectable	24.3
+MCC	3	40.2	23.9
no Ag	1	not detectable	25.6
no Ag	2	not detectable	24.7
no Ag	3	38.3	25.3

Suppl. Figure S1. **(A)** Two-color contour plot illustrating the co-expression of acquired I-Ek and CD80 or acquired I-Ek and CD86 by AND T cells incubated overnight BMDCs loaded or not with MCC antigen. Bar plots to the right shows the mean ± sem of triplicate cultures Statistical analysis was carried out using an unpaired two-tailed Student's t-test. ****p < 0.0001. **(B)** RT-qPCR analysis of CD80 and CD3e gene transcripts of AND T cells stimulated for 2 hours with DCEK APCs loaded or nor with MCC antigen and FACS-sorted. Cq values are given for each biological replica. **(C)** Time-dependent expression of CD86 by AND WT and AND *Rhog*$^{-/-}$ CD4 T cells upon incubation with BMDCs loaded with 10 µg of antigenic MCC peptide. Data points shows the mean ± sem of duplicate cultures. Both, the percentage of I-Ek and CD86 double positive cells and the MFI of CD86 are shown. Statistical analysis was carried out using a paired two-tailed Student's t-test. *p < 0.05. **(D)** I-E^k expression after incubation with DCEK fibroblasts was analyzed by pre-embedding immunogold electron microscopy after labelling with 10 nm streptavidin-gold particles. Blue arrows point at the presence of gold particles associated to the plasma membrane; red arrow to gold particles associated to surface-bound microvesicles. **(E)** Confocal microscopy images of MHC-II complexes acquired by WT AND T cells after one hour of incubation with murine DCEK fibroblasts, transfected with the GFP-tagged I-E^k α subunit. Cells were stained with LAMP1 (Lysosomal-associated membrane protein 1) and CD63, a marker of intracellular vesicles. A confocal mid-plane section is shown. Scale bar = 5 µm. **(F)** CD4 T cells acquire cognate MHC-II complexes together with bystander ones. OT2 T cells were incubated for 2 hours with BMDCs from mice of mixed k/b background loaded with OVAp or with no antigen and analysed by flow cytometry for the expression on their cell surface of cognate I-Ab and bystander I-Ek. Likewise, AND T cells from b/b mice were incubated for 2 hours with BMDCs of k/b background loaded with MCC peptide or no antigen. A representative experiment out of three is shown. Related to Figure 1.

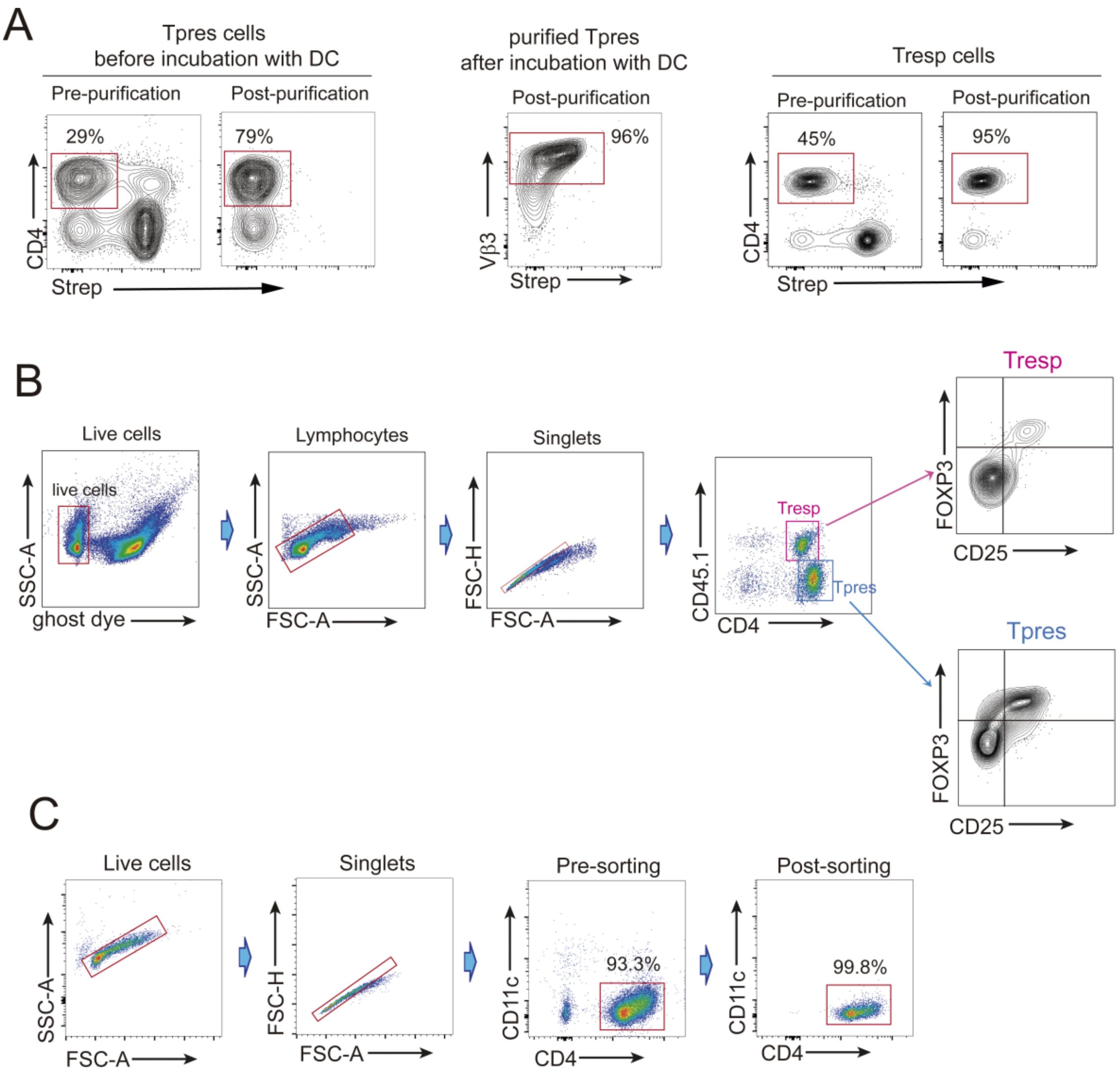

Suppl. Figure S2. (A) Negative selection of T cells. Purity of CD45.2+ AND T cells before and after negative selection using a cocktail of biotinylated antibodies against the following markers: CD11b, CD11c, B220, NK1.1, Gr1 and F4/80. Staining with streptavidin-PerCP and anti-CD4-PE revealed a 79% purity of the CD4+ population. This population was incubated with MCC-loaded BMDCs overnight.After overnight incubation with BMDCs, the CD45.2+ Tpres population was purified again by negative selection as before. Staining with streptavidin-PerCP and anti-Vb3-PE revealed a 96% purity of the AND T cell population. Note that remaining cells were not CD11c+ since a biotintylated CD11c antibody was in the staining mix.Purity of the CD45.2+CD45.1+ Tresp cells before and after negative selection and before co-incubation with Tpres cells. **(B)** Gating strategy for Tresp and Tpres cells in co-culture. Seven-day co-cultures of Tpres and Tresp cells as illustrated in the cartoon of Fig. 3A are first selected for live cells using the ghost dye. Subsequently, lymphocytes are selected within the live cell population according to forward and side scatter. Single cells are then separated from cell doublets according to Forward Scatter-A and Forward Scatter-H. Subsequently, single cell lymphocytes are separated into Tpres and Tresp cells according to the expression of CD4 and the differential marker CD45.1. Finally, expression of Treg markers, or Th17 markers, is analysed within the gated Tpres and Tresp populations. **(C)** Purity of Tpres cells before and after cell sorting. After overnight incubation with antigen-loaded BMDCs, CD45.2+ Tpres cells were removed from the plate and purified by FACS-sorting using CD11c as a DC marker. Note that the pre-sorted population was poor in CD11c+ DCs because most of the DCs remained attached to the plate. After sorting, the CD4+ population was 99.8% pure. Related to Figures 2 and 3.

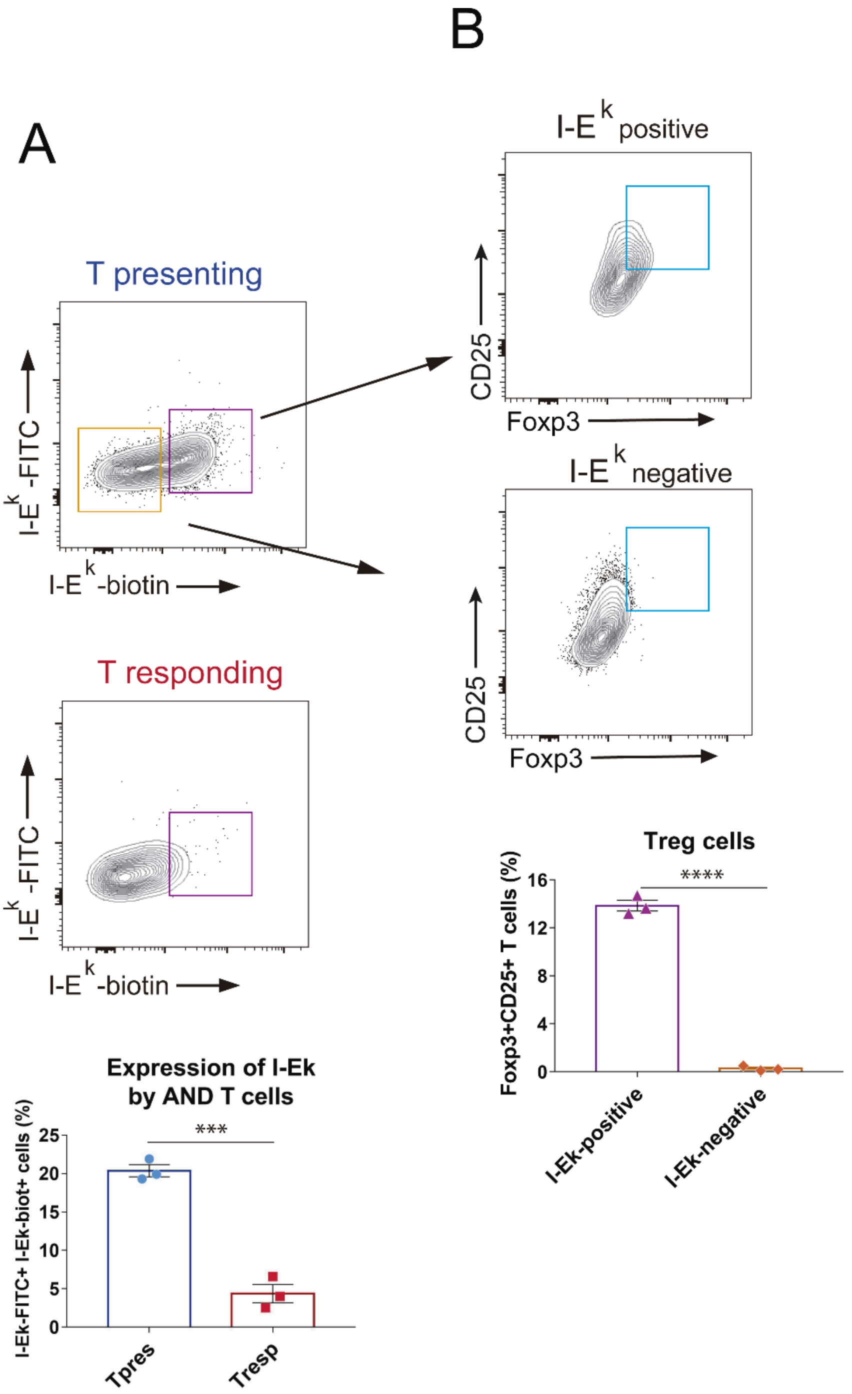

Suppl. Figure S3. **Expression of Treg markers by MHC-II+ trogocytic T cells. (A)** AND T cells from b/b mice were cultured overnight with MCC-loaded BMDCs and then purified and cultured together with naïve AND T cells (Tresp) for 6 days. Tpres and Tresp cells (according to the expression of CD45.1 and CD45.2 markers) were analysed for extracellular I-Ek. Quantification is provided in the bar plot below. Data represent the mean±sem of biological triplicates. ***p <0.001 (two-way unpaired Student's t-test). **(B)** Gated I-Ek-positive and I-Ek-negative Tpres cells in (A) were analysed for expression of extracellular CD25 and intracellular Foxp3. Quantification is provided in the bar plot below. Data represent the mean±sem of biological triplicates. ****p <0.0001 (two-way unpaired Student's t-test). Related to Figure 3.

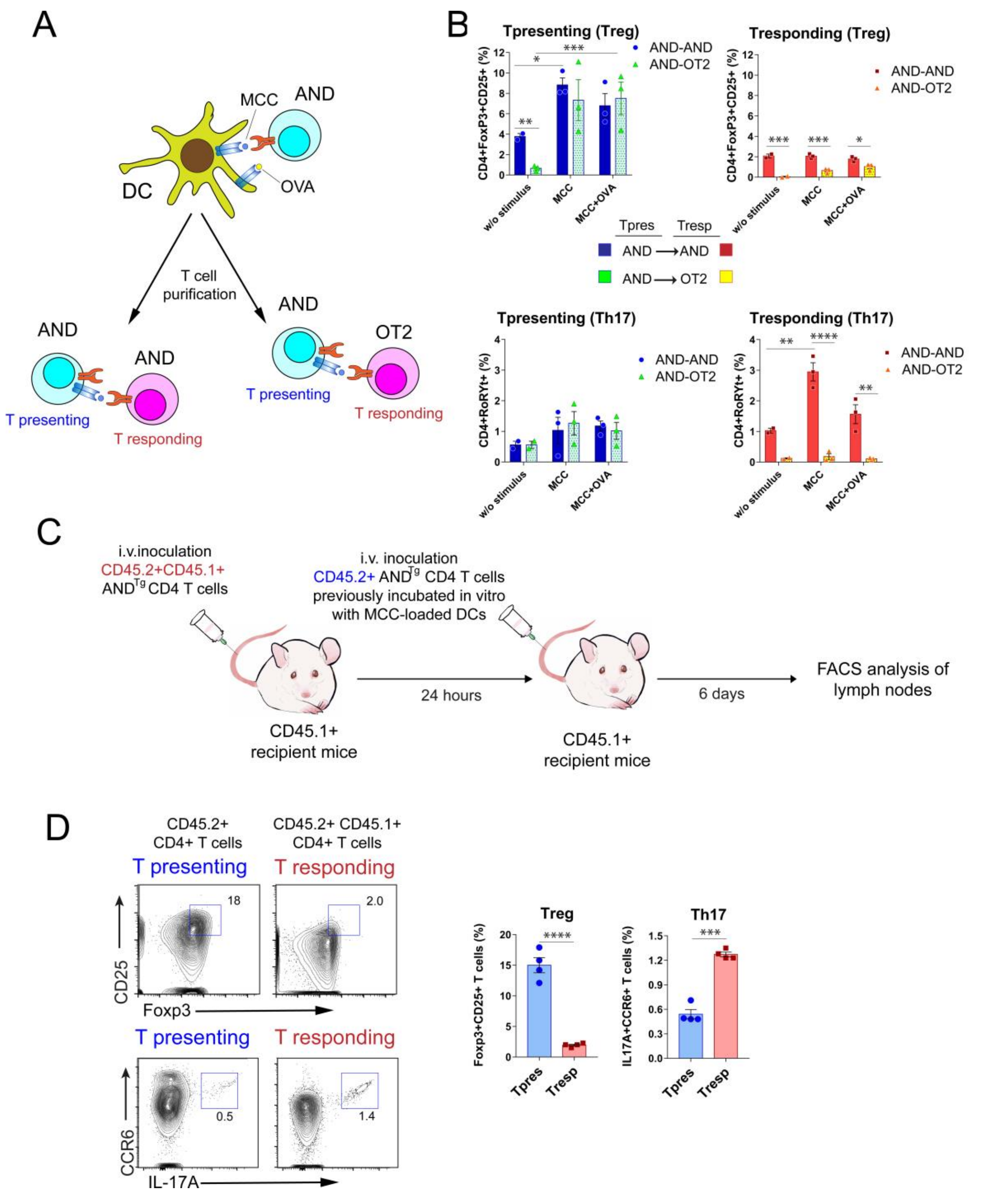

Figure S4. Antigen specificity and *In vivo* differentiation of Tpres into Treg and Tresp into Th17. (A) Experimental set-up**.** BMDCs from mice of mixed k/b background were loaded with MCC and OVA and incubated overnight with AND T cells. After purification, AND Tpres cells were incubated either with AND or OT2 Tresp cells. **(B)** After 6 days of co-culture, AND Tpres and AND or OT2 Tresp cells were stained with surfaceCD25 and intracellular Foxp3 or RoRγt. Bar plots represent the mean±sem of biological triplicates. **p < 0.01; ****p <0.0001; ns, not significant (two-way ANOVA test). Two color contour plots are on the left and quantification on the bar plots to the right. *p < 0.05; **p < 0.01; ***p <0.001; ****p <0.0001 (two-tailed unpaired Student's *t*-test). **(C)** Experimental set-up**.** Tpres and Tresp cells in lymph nodes were identified according to the expression of CD45.1 and CD5.2 alleles. **(D)** Two-color contour plot analysis of Treg marker (CD25 and Foxp3) and Th17 marker (IL-17A and CCR6) expression in CD4+ Tpres and Tresp cells. Quantification is shown in the bar plots as mean ± sem. (n=4 mice per group). ***p < 0.001, ****p <0.0001 (two-tailed unpaired Student's *t*-test). Related to Figures 2 and 3.

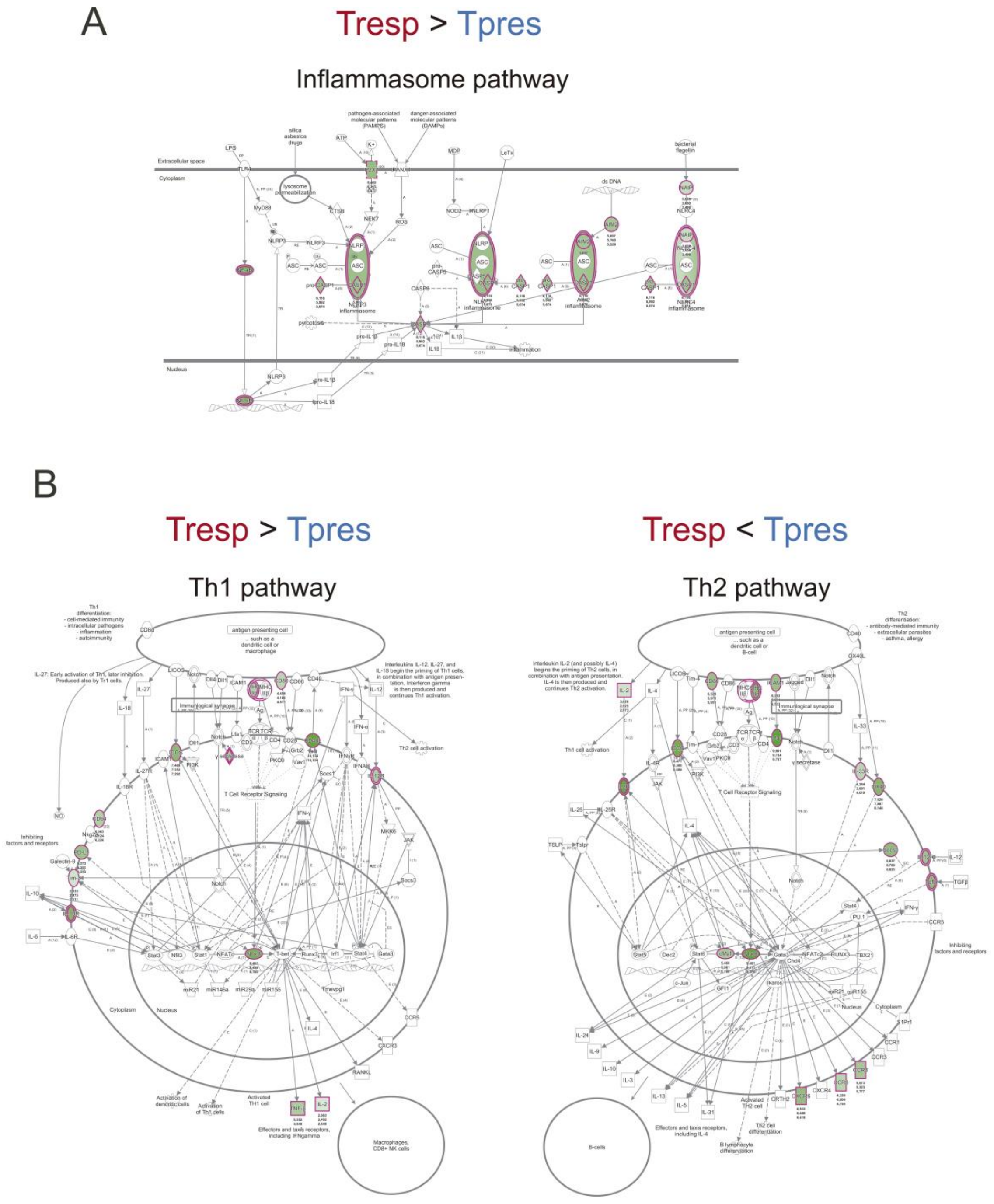

Suppl. Figure S5. **IPA Analysis of Tpres and Tresp cells.** The top score IPA Inflammasome **(A)** and Th1 **(B)** pathways are mainly expressed by Tresp and the IPA Th2 pathway is more expressed by Tpres. Different shapes represent the molecular classes of the proteins: kinases are shown as triangles, membrane receptors as double ellipses, transcriptional regulators as single ellipses, cytokines and chemokines as squares. Direct and indirect interactions are indicated by solid and dashed lines, respectively. Related to Figure 4.

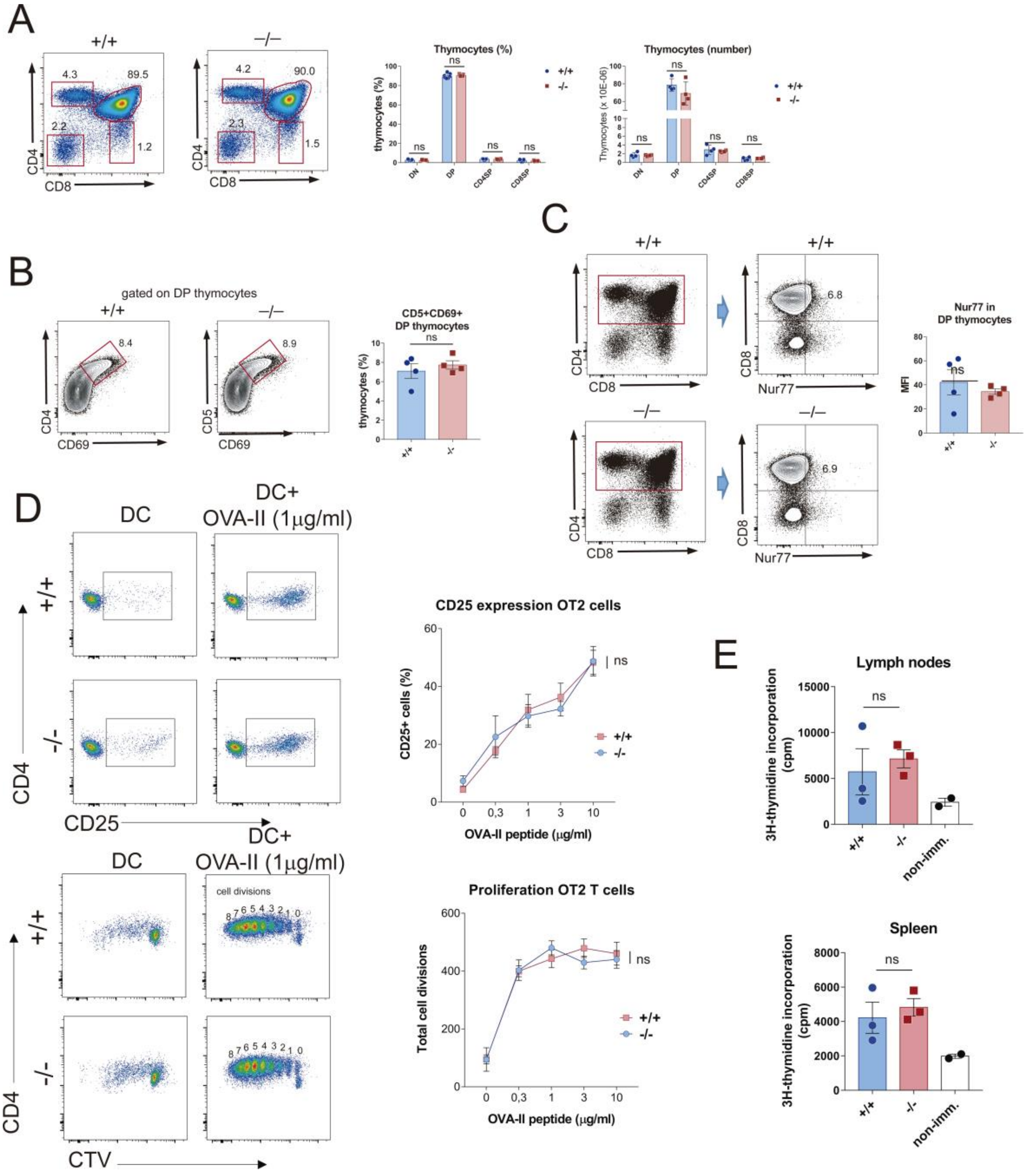

Suppl. Figure S6. **Phenotypic and functional analysis of *Rhog*$^{-/-}$ mice. (A)** Major thymic populations (DN: CD4-CD8-, DP: CD4+CD8+, CD4SP: CD4+CD8-, CD8SP: CD4-CD8+) in 8-week-old WT and *Rhog*$^{-/-}$ mice. Percentage and total cell number for n=4 mice per genotype are given in the bar plots. ns, not significant (two-way unpaired Student's t-test). **(B)** Expression of CD5 and CD69 within the DP population gated as in (A) in 8-week-old WT and *Rhog*$^{-/-}$ mice. Bar plot on the right shows the mean ± sem (n=4 mice per group) ns, not significant (two-way unpaired Student's t-test). **(C)** Expression of the TCR strength-dependent marker Nur77 in DP thymocytes of 8-week-old WT and *Rhog*$^{-/-}$ mice. Bar plot on the right shows the mean ± sem (n=4 mice per group). ns, not significant (two-way unpaired Student's t-test). **(D)** Activation of OT2 WT and OT2 *Rhog*$^{-/-}$ T cells in response to BMDCs loaded with different doses of OVAp was analysed by CD25 expression after a 24 h incubation and by T cell proliferation measured according to Cell Trace Violet (CTV) dilution after 72 h of incubation. Cell divisions are indicated on top of the CTV pseudocolor plot. Line plots show the mean ± sem of triplicate biological samples. ns, not significant (two-way paired Student's t-test). **(E)** Proliferation of CD4 T cells from draining popliteal lymph nodes and spleen from WT and *Rhog*$^{-/-}$ MOG-immunized mice in response to stimulation ex vivo with BMDCs loaded with MOG was quantitated by 3H-thymidine incorporation. The bar plot shows the mean ± sem (n=3 mice per immunized group). T cells stimulated ex vivo with MOG from two non-immunized mice were used as controls of background proliferation. ns, not significant (two-way unpaired Student's t-test). Related to Figure 5.

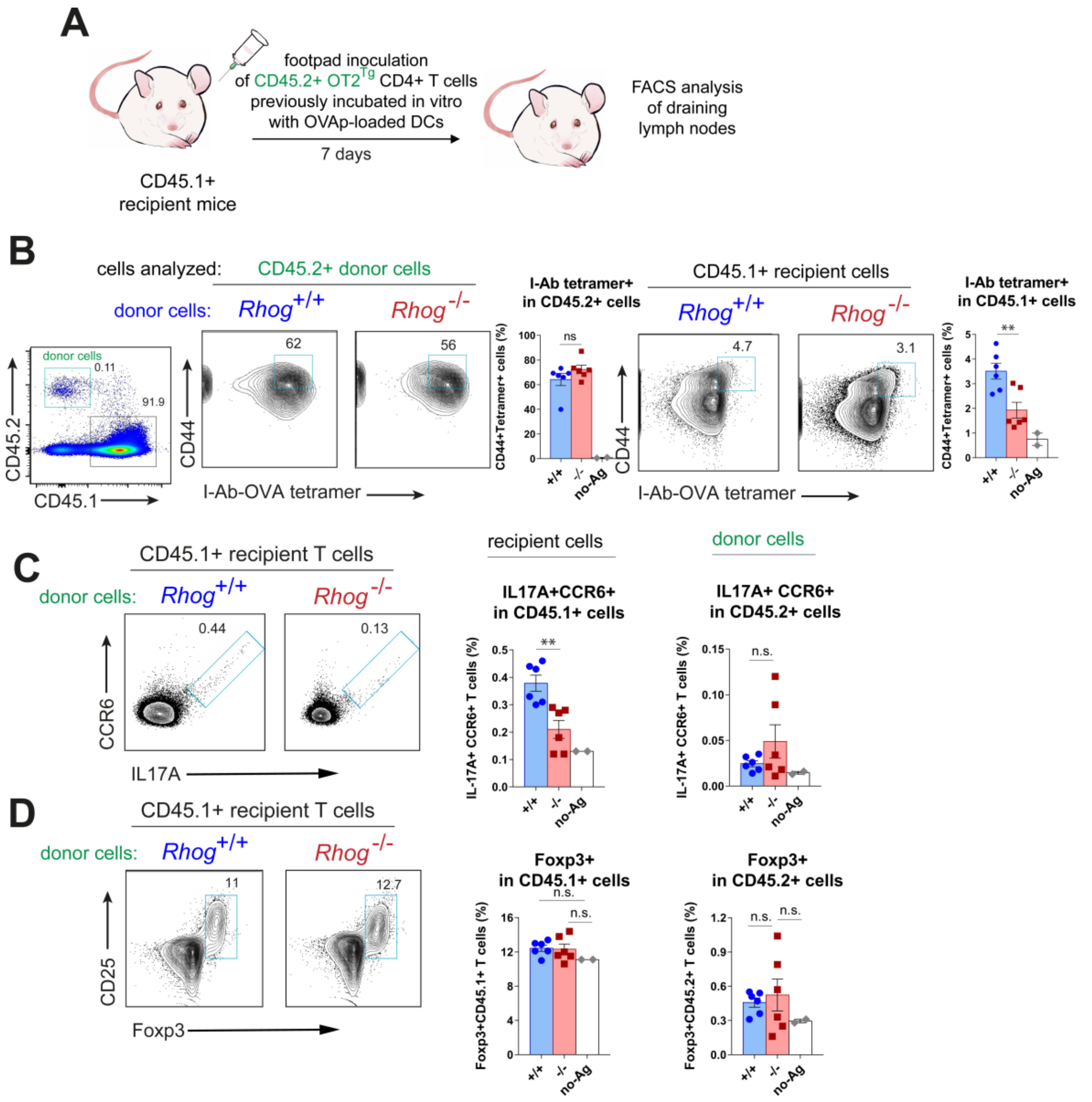

Suppl. Figure S7. **Tpres cells can activate Tresp cells of the endogenous polyclonal T cell repertoire.** **(A)** Experimental set-up. CD45.1^{+} as recipient mice were inoculated by footpad injection with 5x10^{6} OT2 CD45.2+CD4+ T cells purified from WT and *Rhog*$^{-/-}$ mice after being incubated overnight with OVAp antigen-loaded DCs. **(B)** Two-color contour plot analysis of I-Ab- (OVAp) tetramer specific CD4 T cells within the antigen-experienced CD44+ populations of donor (CD45.2+) and recipient's (CD45.1+) cells. Quantification is shown in the bar plots as mean± sem (n=6 mice per group). No-Ag, data generated in mice inoculated with OT2 CD45.2+CD4+ T cells from WT mice incubated overnight with DCs not loaded with antigen. ** p < 0.01, ns not significant (two-tailed unpaired Student's *t*-test). **(C, D)** Two-color contour plot analysis of Th17 marker (IL-17A, CCR6) and Treg marker(Foxp3, CD25) expression within the recipient's CD45.1 CD4 T cell population donor cells after inoculaton of WT or *Rhog*$^{-/-}$ OT2 CD4 T cells. Quantification is shown in the bar plots as mean± sem (n=6 mice per group). ** p < 0.01, ns not significant (two-tailed unpaired Student's *t*-test). Related to Figures 3 and 5.

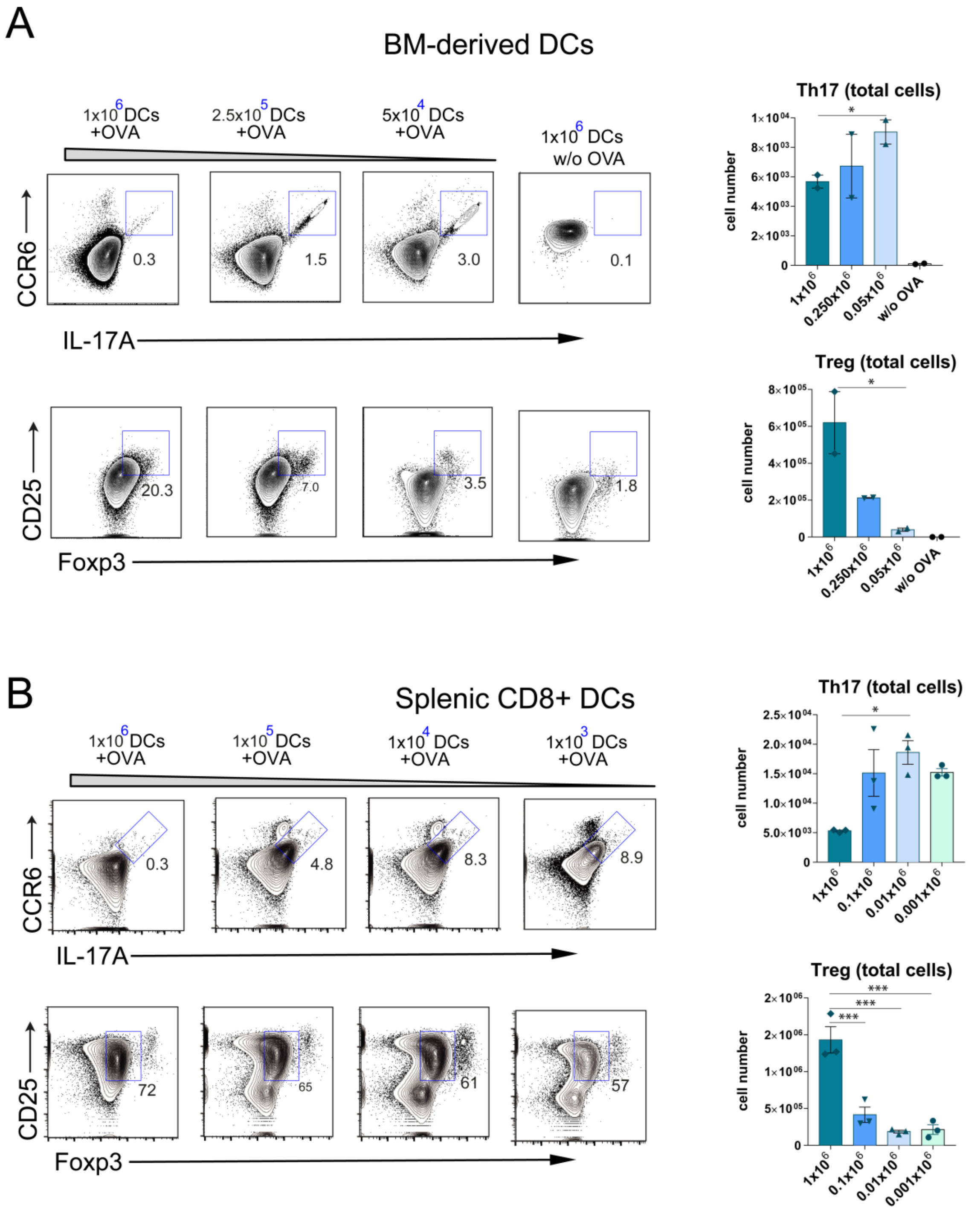

Suppl. Figure S8. **The DC:T cell ratio determines Treg vs Th17 differentiation *in vitro*.** A constant number of OT2 CD4 T cells (2.5 x10^6) was co-incubated for 6 days with varying numbers of either BM-derived DCs (**A**) or purified splenic CD8+ DCs (**B**) loaded with OVAp antigen. Treg vs Th17 differentiation was analyzed according to the expression of CD25, Foxp3, IL-17A and CCR6 markers. Bar plots show the mean ± sem of n= 2-3 replicas. Total number of cells with the Treg and Th17 phenotype are shown. * $p < 0.05$; *** $p < 0.001$ (two-tailed unpaired Student's *t*-test). Related to Figure 6.

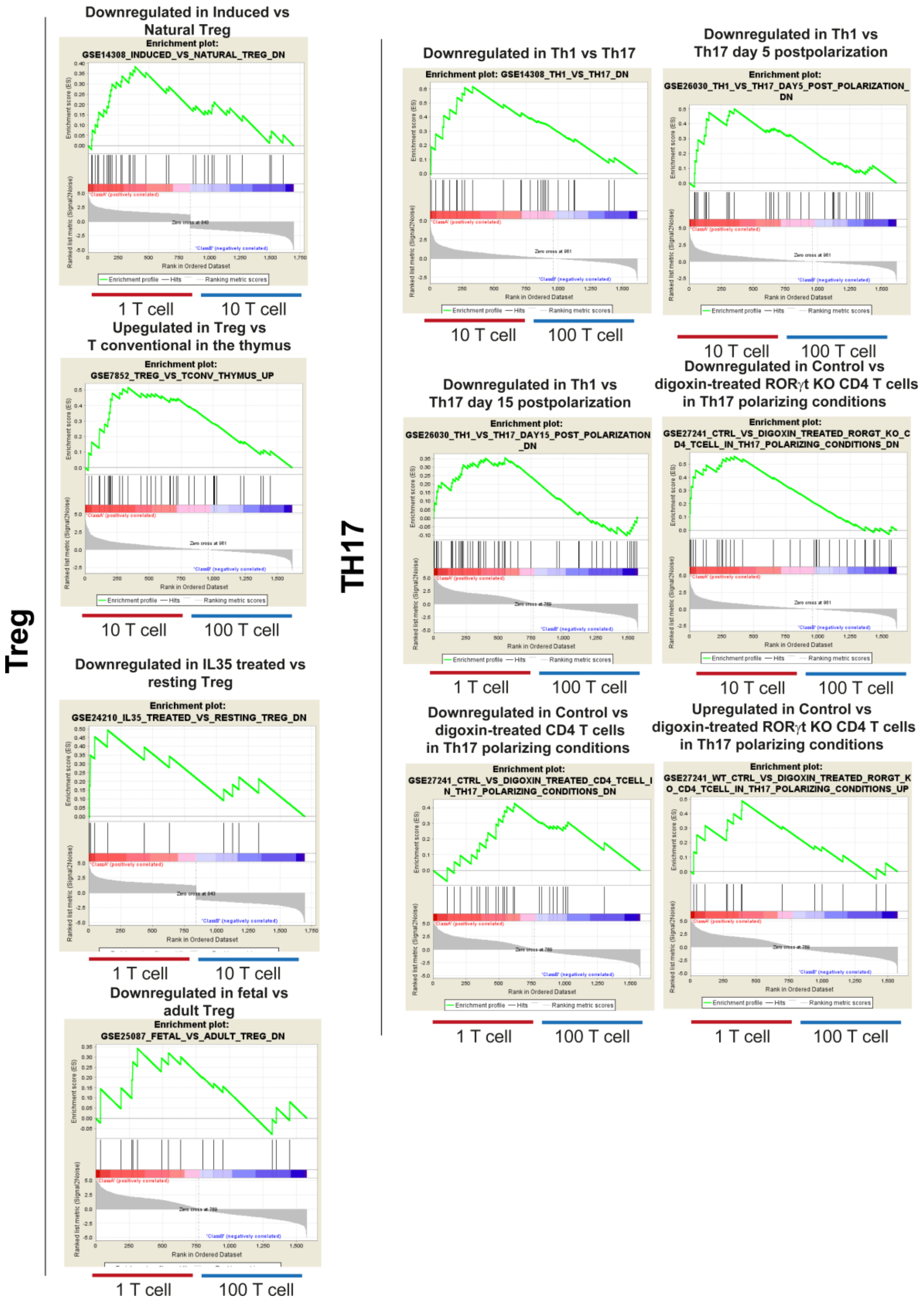

Suppl. Figure S9. Low T cell: DC ratio favor Treg whilst high ratios favor Th17. Gene set enrichment analysis (GSEA) of mRNA gene expression in the culturing conditions of 1 T cell/well, 10 T cells/well and 100 T cells/well (Fig. 7B) analyzed using the Broad Institute's GSEA mSig database. Related to Figure 6.
